# CDC7 Inhibition Potentiates Antitumor Efficacy of PARP Inhibitor in Advanced Ovarian Cancer

**DOI:** 10.1002/advs.202403782

**Published:** 2024-10-16

**Authors:** Shini Liu, Peng Deng, Zhaoliang Yu, Jing Han Hong, Jiuping Gao, Yulin Huang, Rong Xiao, Jiaxin Yin, Xian Zeng, Yichen Sun, Peili Wang, Ruizi Geng, Jason Yongsheng Chan, Peiyong Guan, Qiang Yu, Bin‐Tean Teh, Qingping Jiang, Xiaojun Xia, Ying Xiong, Jianfeng Chen, Yongliang Huo, Jing Tan

**Affiliations:** ^1^ State Key Laboratory of Oncology in South China Guangdong Provincial Clinical Research Center for Cancer Sun Yat‐sen University Cancer Center Guangzhou Guangdong 510060 P. R. China; ^2^ Guangdong Provincial People's Hospital Guangdong Academy of Medical Sciences School of Medicine Southern Medical University Guangzhou Guangdong 510080 P. R. China; ^3^ Biotherapy Center Sun Yat‐Sen Memorial Hospital Sun Yat‐Sen University Guangzhou 510120 P. R. China; ^4^ Guangdong Provincial Key Laboratory of Colorectal and Pelvic Floor Diseases The Sixth Affiliated Hospital of Sun Yat‐sen University Guangzhou Guangdong 510655 P. R. China; ^5^ Cancer and Stem Cell Biology Program Duke‐NUS Medical School Singapore 169857 Singapore; ^6^ Department of Laboratory Medicine Guangzhou First People's Hospital School of Medicine South China University of Technology Guangzhou 510180 P. R. China; ^7^ Experimental Animal Center Guangzhou Municipal and Guangdong Provincial Key Laboratory of Protein Modification and Degradation School of Basic Medical Sciences Guangzhou Medical University Guangzhou 511436 P. R. China; ^8^ Division of Medical Sciences Laboratory of Cancer Epigenome National Cancer Centre Singapore Singapore 169610 Singapore; ^9^ Genome Institute of Singapore A*STAR Singapore 138672 Singapore; ^10^ Department of Patholgy Guangdong Provincial Key Laboratory of Major Obstetric Disease The Third Affiliated Hospital of Guangzhou Medical University Guangzhou Guangdong 510150 China; ^11^ Hainan Academy of Medical Science Hainan Medical University Haikou 571199 P. R. China

**Keywords:** CDC7 inhibitor, immune activation, ovarian cancer, PARP inhibitor resistance, target therapy

## Abstract

Poly (ADP‐ribose) Polymerase inhibitors (PARPi) have demonstrated remarkable clinical efficacy in treating ovarian cancer (OV) with BRCA1/2 mutations. However, drug resistance inevitably limits their clinical applications and there is an urgent need for improved therapeutic strategies to enhance the clinical utility of PARPi, such as Olaparib. Here, compelling evidence indicates that sensitivity of PARPi is associated with cell cycle dysfunction. Through high‐throughput drug screening with a cell cycle kinase inhibitor library, XL413, a potent cell division cycle 7 (CDC7) inhibitor, is identified which can synergistically enhance the anti‐tumor efficacy of Olaparib. Mechanistically, the combined administration of XL413 and Olaparib demonstrates considerable DNA damage and DNA replication stress, leading to increased sensitivity to Olaparib. Additionally, a robust type‐I interferon response is triggered through the induction of the cGAS/STING signaling pathway. Using murine syngeneic tumor models, the combination treatment further demonstrates enhanced antitumor immunity, resulting in tumor regression. Collectively, this study presents an effective treatment strategy for patients with advanced OV by combining CDC7 inhibitors (CDC7i) and PARPi, offering a promising therapeutic approach for patients with limited response to PARPi.

## Graphical abstract

The study shows that resistance to PARP inhibitors in ovarian cancer correlates with aberrant cell cycle‐related pathways. Inhibition of cell cycle‐related proteins CDC7 and Olaparib synergistically induces significant DNA damage and DNA replication stress and activates the cGAS/STING signaling pathway, resulting in remarkable antitumor immunity effects. This provides a promising treatment strategy for PARP inhibitor‐resistant ovarian cancer.

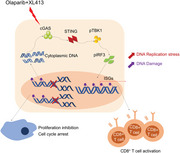

## 1 Introduction

Ovarian cancer (OV) remains a major cause of gynecologic cancer‐related mortality in women. OV is frequently diagnosed at advanced stages with limited effective therapeutic options available.^[^ ^1^ ^]^ ≈50% of OV cases have highly unstable genomes, which is characterized by homologous recombination deficiency (HRD), accompanied with mutations in homologous recombination repair (HRR)‐related genes such as *BRCA1/2*, offering a potential avenue for treatment with PARPi.^[^ ^2^ ^]^ Currently, a variety of PARPi have been approved for use in the maintenance therapy and in the relapsed setting of OV.^[^ ^3^ ^]^ However, the issue of intrinsic and acquired resistance to PARPi remains a significant challenge, leading to suboptimal clinical outcomes.^[^ ^4^ ^]^

Extensive research efforts have focused on exploring the various mechanisms underlying resistance to PARPi, including the restoration of homologous recombination (HR) signaling through genetic mutations or epigenetic alterations, dysfunction of PARP enzymes, DNA replication stress, and over‐expression of drug efflux pumps.^[^ ^5^ ^]^ Strategies aimed at inducing HRD or targeting HR genes exhibit promising results in improving the efficacy of PARPi via synthetic lethality.^[^ ^6^, ^7^ ^]^ Fork protection and DNA replication stress resulting in resistance to PARPi have been reported in BRCA1/2‐deficient cells. Deubiquitinase USP1 directly bound to and stabilized the replication fork in BRCA1‐deficient cells, which protected cancer cells from PARPi.^[^ ^8^ ^]^ PBRM1 deletion caused synthetic lethality to PARPi by inducing accumulation of R‐loop under conditions of replication stress and DNA damage.^[^ ^9^ ^]^ Overexpression of ABCB1, the first drug efflux transporter, has been proposed to induce resistance to PARPi in BRCA1/2‐deficient tumor.^[^ ^10^ ^]^ Thus, identifying the key molecular events associated with PARPi resistance will allow for the development of new combinational therapeutic options, which are essential for improving survival rates of OV patients. PARPi have been observed to impact antitumor immune responses through different mechanisms, including activation of cGAS/STING and induction of immune response through DNA damage and genome instability.^[^ ^11^ ^]^ Combined inhibition of PARP and checkpoint kinase 1 (CHK1) has also demonstrated enhanced antitumor effects in immune checkpoint blockade therapy (ICB) and increased cytotoxic T‐cell infiltration.^[^ ^12^ ^]^ Combined treatment of PARPi and immunotherapy blockades is a growing potential approach to the treatment of patients with BRCA1/2‐mutant cancer.^[^ ^13^, ^14^ ^]^

While the combination of PARPi with immunotherapy holds potential for BRCA1/2‐mutant cancers, limited benefits have been reported in recent studies,^[^ ^15^ ^]^ highlighting the need for alternative strategies to potentiate antitumor immunity and immunotherapy response. In this context, we propose a novel combination therapeutic strategy with CDC7i to sensitize resistant OV to PARPi, leading to cell cycle arrest and DNA damage response. Importantly, our study demonstrates that this combination boosts cGAS/STING pathway to activate interferon response signaling, thereby potentiating antitumor immunity. Thus, this novel combination strategy represents an exciting avenue for improving clinical outcomes and provides optimism for overcoming resistance to PARPi in OV management.

## 2 Results

### 2.1 Olaparib Sensitivity is Associated with Cell Cycle Dysfunction

**Figure 1:**
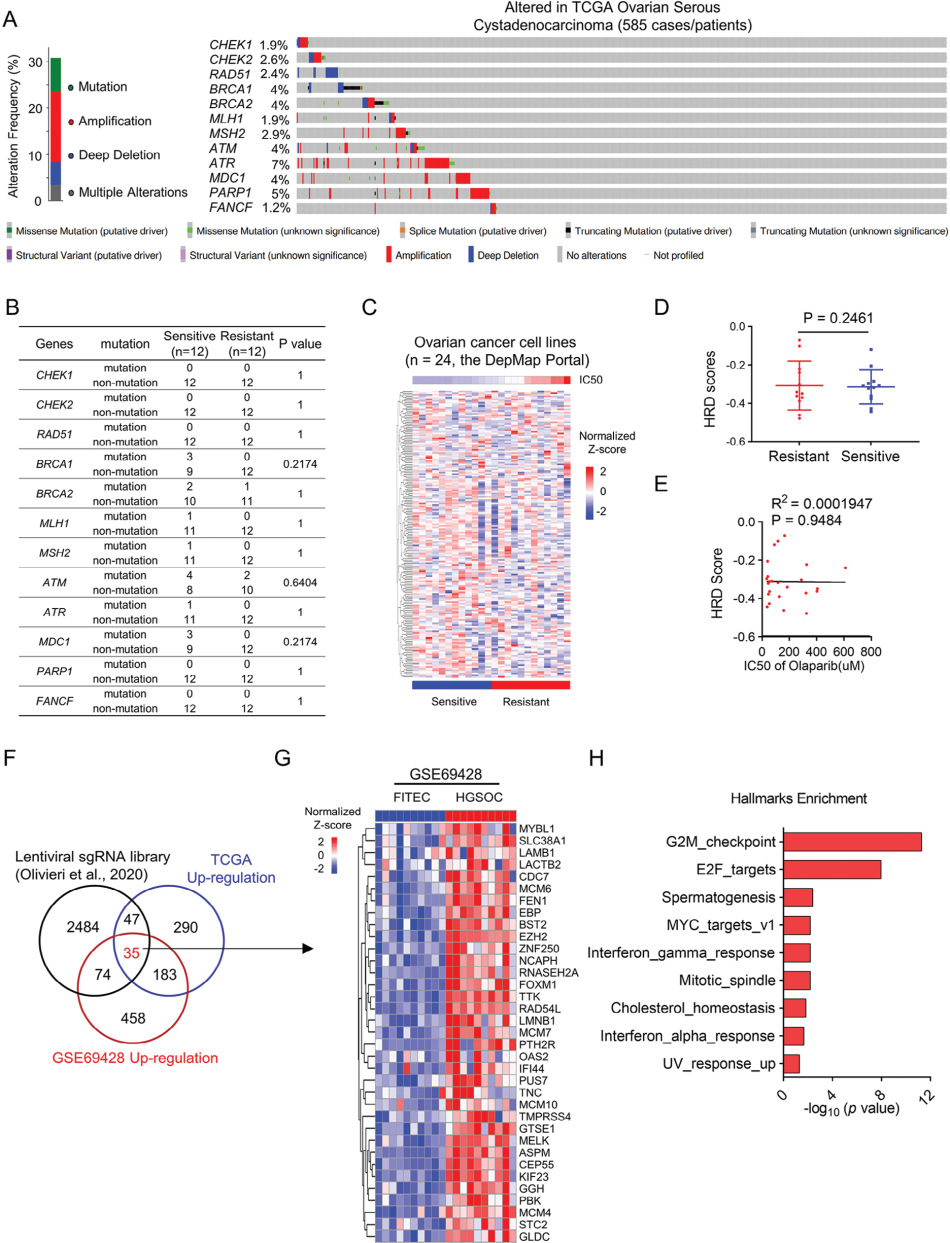
Olaparib sensitivity is associated with cell cycle dysfunction. A) Genetic alterations including mutations and copy number alterations of DNA damage response genes (*CHEK1, CHEK2, RAD51, BRCA1, BRCA2, MLH1, MSH2, ATM, ATR, MDC1, PARP1, FANCF*) in TCGA ovarian serous cystadenocarcinoma (n = 585, data were downloaded from cBioPortal, http://www.cbioportal.org). B) Fisher exact test was used for the association between the mutation of DNA damage response genes and Olaparib IC50 of 24 OV cell lines from the GDSC datasets. C,D) Heatmap (C) and HRD scores (D) from unsupervised clustering of HRD gene signatures using transcriptome data of 24 OV cell lines from the DepMap Portal. Data are shown as mean ± SD (two‐tailed *t*‐test). E) Correlation between HRD scores and IC50 values of 24 OV cell lines from the CCLE database. Statistical correlations were analyzed by the Pearson correlation coefficient. F) Venn diagram showing the overlap of regulators of Olaparib identified by CRISPR screens, genome‐wide transcriptional data from TCGA‐OV (TCGA‐OV transcriptome_U133A‐seq database), and upregulated genes in OV from GEO dataset (GSE69428). G) Heatmap of differentially expressed genes (*p* < 0.05) in a cohort of HGSOCs (n = 10) compared with matched normal oviduct samples (n = 10) from the GSE69428 dataset. H) The top enriched pathways were shown using the overlapped 35 genes from Figure 1F.

Although Olaparib is primarily used in advanced OV patients harboring mutated BRCA1/2, emerging evidence suggests that not all patients with BRCA1/2 mutation are sensitive to PARPi^[^ ^5^, ^16^ ^]^ and some patients without these mutations may also show sensitivity to PARPi.^[^ ^17^, ^18^ ^]^ To investigate the correlation between the sensitivity to PARPi (Olaparib, Niraparib, and Talazoparib) and somatic mutations in DNA damage‐related genes, we first examined the mutation status of DNA damage response genes in ovarian serous cystadenocarcinoma from TCGA database (PanCancer Atlas) through cBioportal (http://www.cbioportal.org/). The data showed that low alteration frequencies were observed in DNA damage response genes (**Figure** **1**A). For example, the alteration frequency of BRCA1 was 4% in this cohort, including seven cases with deep deletion and 18 cases with mutation. The frequency of ATR is relatively higher than other genes, while the mutation rates were only 7%. In addition, we also examined the mutational status of BRCA1/2 of OV cells and half‐maximal growth‐inhibitory concentration (IC50) data of PARPi (Olaparib, Niraparib, and Talazoparib) downloaded from the Genomics of Drug Sensitivity in Cancer dataset (GDSC, https://www.cancerrxgene.org/). The data showed no significant difference in PARPi sensitivity between wild‐type and BRCA1‐mutant OV cells (Figure S1A, Supporting Information). Subsequently, we defined 24 OV cell lines into sensitive and resistant cells according to the IC50 data (Figure S1B, Supporting Information) and examined the mutation status of DNA damage response genes in OV cells from the DepMap Portal. These results indicated that no significant correlation between the sensitivity to PARPi and the mutation status of each single DNA repair genes (Figure 1B). Previous studies have reported that the HRD score derived from the transcriptional expression of a specific set of genes could predict the sensitivity of PARPi in OV.^[^ ^19^ ^]^ Therefore, we assessed the transcriptional profiling data of the OV cells and calculated the HRD gene signature (Figure 1C). These results suggested that there was no significant correlation between the HRD gene signature and sensitivity to Olaparib (Figure 1D,E). Furthermore, using loss‐ and gain‐of‐function studies, our findings indicated that BRCA1 status did not change the sensitivity of the cells to Olaparib (Figure S1C–H, Supporting Information). In addition, we constructed acquired PARPi‐resistant cells from OVCAR3 cells, which are intrinsically sensitive to PARPi and lack BRCA1/2 mutations. We found no significant difference in HR repair efficiency and HRR genes expression levels between acquired resistant and sensitive cells (Figure S1I–M, Supporting Information). These provide additional evidence supporting our hypothesis that BRCA1/2 mutations and HR status do not fully explain sensitivity to PARPi. It is imperative to investigate novel effective measures to overcome PARPi resistance.

To gain further insights into the molecular basis of Olaparib sensitivity, we performed an integrative analysis using data from i) Clustered regularly interspaced short palindromic repeats (CRISPR) screens for Olaparib regulators,^[^ ^20^ ^]^ ii) upregulated genes in the OV tissue versus normal tissue from the TCGA data and iii) upregulated genes in the OV compared with matched normal oviduct samples (GSE69428) (n = 10).^[^ ^21^ ^]^ Among the overlapping genes, 35 were significantly upregulated and considered as potential negative regulators of Olaparib sensitivity (Figure 1F,G). Pathway analysis of these 35 genes using Hallmark gene sets highlighted significant enrichment of cell cycle‐related pathways, including G2/M checkpoint genes, E2F targets, and MYC targets (Figure 1H). This observation strongly suggested that dysregulation of cell cycle‐related pathways may play a crucial role in determining Olaparib sensitivity in OV. In addition, the Gene Set Enrichment Analysis (GSEA) using Hallmark sets further supported this notion that cell cycle‐related pathways were downregulated in sensitive cells (Figure S1N, Supporting Information). We found the same results in another PARP‐sensitive and acquired resistance dataset (Figure S1O,P, Supporting Information), where cell cycle‐related genes and pathways were upregulated in the resistant group. Collectively, these findings provide compelling evidence that targeting cell cycle‐related pathways may enhance Olaparib sensitivity in OV.

### 2.2 Drug Screening Identifies CDC7 Inhibitor as a Potential Sensitizer to Olaparib in OV

**Figure 2:**
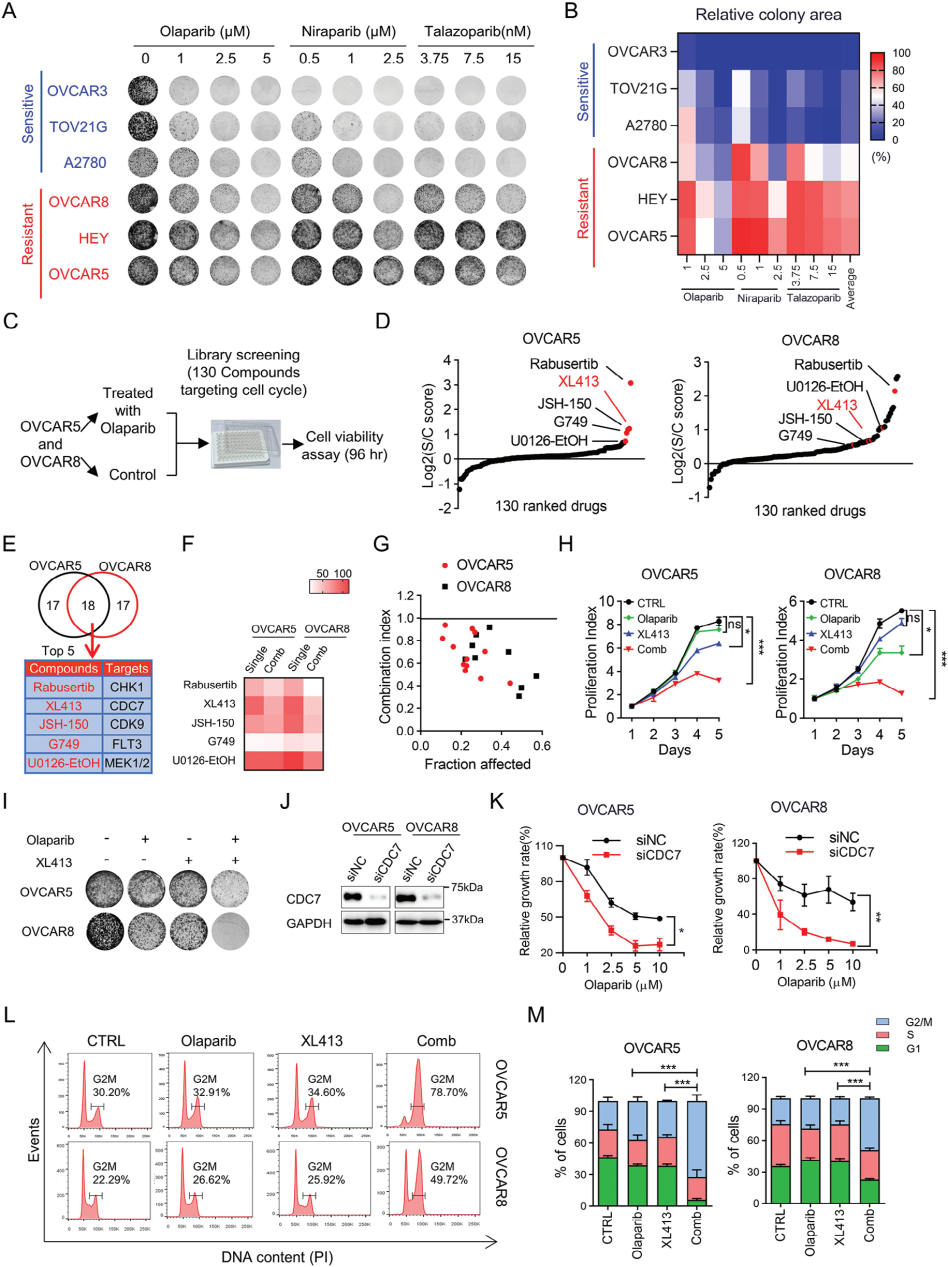
Drug screening identifies CDC7 inhibitor as a potential sensitizer to Olaparib in OV. A,B) Representative images (A) and quantifications (B) of colony formation assay in OV cell lines treated with three PARPi (Olaparib, Niraparib, and Talazoparib) at indicated concentrations. The cut‐off values for sensitive and resistant were 50% based on the relative colony area (in comparison to the control group). C) Outline of the drug screening using 130 kinase inhibitors that target cell cycle‐related pathways in combination with Olaparib in OVCAR5 and OVCAR8 cells. D) Distribution of the S/C (single/combination) score for all 130 kinase inhibitors in OVCAR5 and OVCAR8 cells upon combinational drug screening. E) The top 5 overlapped drugs were identified as sensitizers of Olaparib in OVCAR5 and OVCAR8 cells. F) Heatmap showing the survival rate of OVCAR5 and OVCAR8 cells treated with the top 5 overlapped drugs (1.0 µm) from the drug screen in the presence or absence of Olaparib. G) Combination index analysis of XL413 and Olaparib in OVCAR5 and OVCAR8 cells (Combination index value < 1 was defined as synergy). H) Cell proliferation assay showing the growth effect of indicated treatments in OVCAR5 and OVCAR8 cells. I) Colony formation assay in OVCAR5 and OVCAR8 cells in response to combination treatment of Olaparib and XL413. J) Immunoblot analysis of CDC7 and GAPDH in lysates collected from OVCAR5 and OVCAR8 cells transfected with siNC or siCDC7. K) Sensitivity of OVCAR5 and OVCAR8 cells transfected with siNC or siCDC7 in response to indicated concentrations of Olaparib. L,M) Representative images (L) and quantifications (M) of the percentages of G2/M, S, and G1 phase in OVCAR5 and OVCAR8 cells. Cells were exposed to indicated treatments, stained with propidium iodide, and analyzed using flow cytometry. Data in (H,K) are shown as mean ± SD of triplicate (two‐way ANOVA). Data in (M) are shown as mean ± SD of triplicate (one‐way ANOVA). ns, no significance, ^*^ *p* < 0.05, ^**^ *p* < 0.01; ^***^ *p* < 0.001.

To determine whether targeting cell cycle‐related pathways could overcome resistance to Olaparib, we treated a panel of OV cell lines with increasing concentrations of Olaparib, Niraparib, and Talazoparib. According to the relative clonogenic potential of the cells in response to the PARPi, we divided these OV cell lines into sensitive and resistant group (**Figure** **2**A,B). We next conducted a drug screening with 130 compounds targeting cell cycle‐related kinases in combination with PARPi in OVCAR5 and OVCAR8 cells which are intrinsically resistant to PARPi (Figure 2C). We identified several inhibitors that exhibited strong combinatorial effects with Olaparib compared to single treatment (Figure 2D). Among these, five targets were identified as highest‐ranking sensitizers of Olaparib in both OVCAR5 and OVCAR8 cells (Figure 2E). Further validation in a secondary screening confirmed the potential of these targets (Figure 2F). The first‐ranked drug target was the CHK1 inhibitor Rabusertib, which has been reported in previous studies.^[^ ^22^ ^]^ The second‐ranked drug target was the CDC7i,  XL413. CDC7, a serine‐threonine kinase, plays a critical role in the initiation of DNA replication by phosphorylating the minichromosome maintenance protein (MCM) complex. CDC7 also regulates the activation of cell cycle replication checkpoints and is involved in the maintenance of DNA replication forks and mediating DNA damage response.^[^ ^23^ ^]^

To further confirm the combination effects of CDC7i XL413 with Olaparib, we performed combination index (CI) analysis in two resistant cell lines and two patient‐derived OV cells (POVC). Based on the Chou–Talalay combination index model, all CI values were found to be less than 1, indicating a strong synergistic effect between XL413 and Olaparib (Figure 2G; Figure S2A, Supporting Information). Additionally, combinatorial treatment with XL413 and Olaparib significantly reduced cell viability and inhibited colony formation in OV cells and acquired resistant cells OVCAR3‐R (Figure 2H,I; Figure S2B–D, Supporting Information). Similar results were obtained when Olaparib was used in combination with another CDC7i, PHA‐767491 (Figure S2E,F, Supporting Information). Furthermore, genetic depletion of CDC7 using small interfering RNA (siRNA) significantly enhanced the anti‐tumor effect of Olaparib in both colony formation and cell proliferation (Figure 2J,K; Figure S2G, Supporting Information), which was further confirmed with short hairpin RNA (shRNA) targeting CDC7 (Figure S2H,I, Supporting Information). Cell cycle analysis by flow cytometry showed that CDC7i combined with Olaparib led to a significant G2/M arrest (Figure 2L,M), which was further confirmed through p‐H3 staining assay (Figure S2J, Supporting Information). Taken together, these data indicated that CDC7i have the potential to enhance the anti‐tumor effect of Olaparib.

### 2.3 Enhanced Genome Instability and Replication Stress by CDC7 Inhibition in Combination with Olaparib

**Figure 3:**
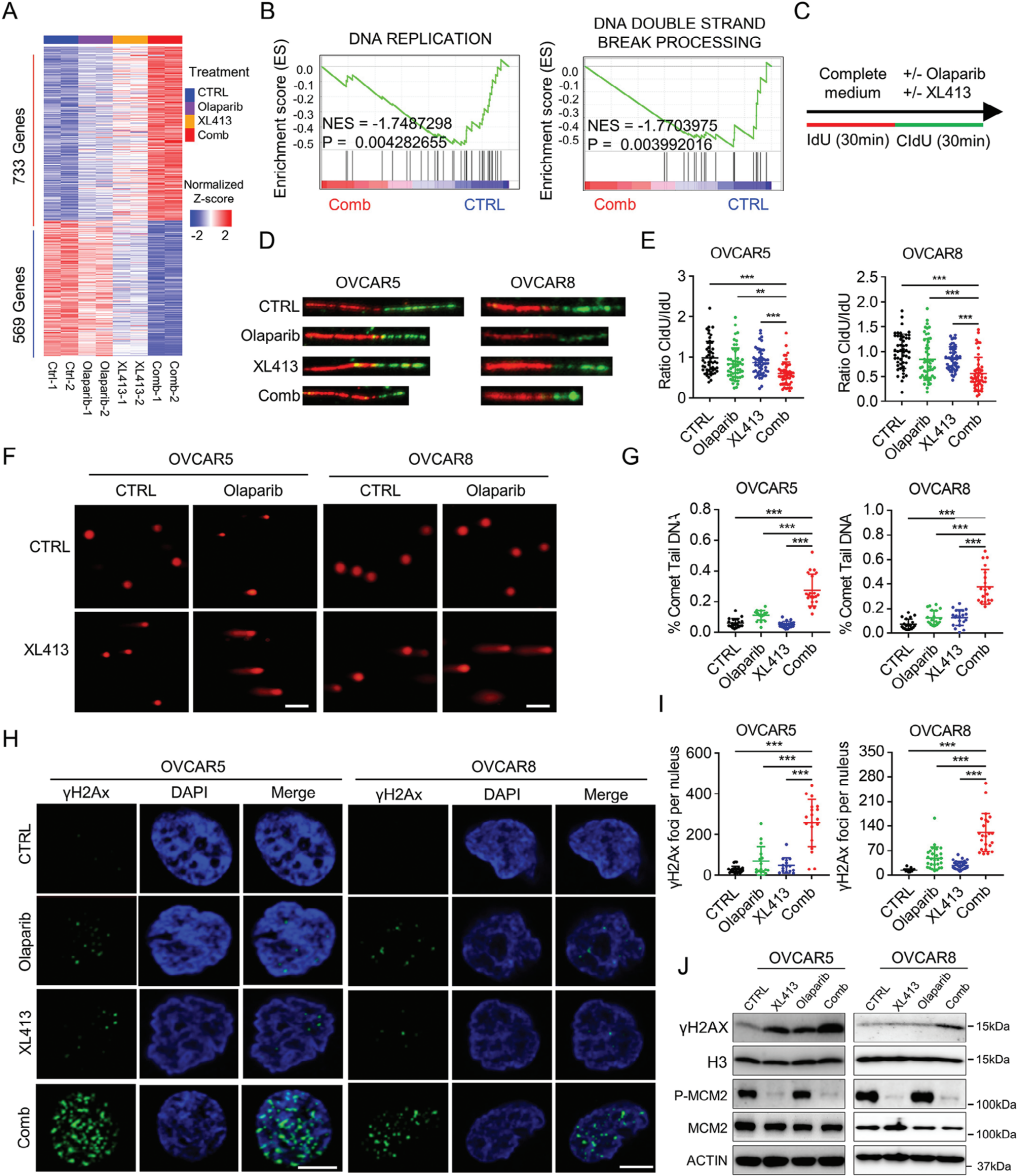
Enhanced genome instability and replication stress by CDC7 inhibition in combination with Olaparib. A) Heatmap of differentially expressed genes (*p* < 0.05) in OVCAR5 cells following the indicated treatments (Duplicates). B) GSEA analysis using RNA‐seq data showed the significant enrichment of the Gene Ontology (GO) gene sets “the DNA REPLICATION” and the KEGG gene sets “DNA_DOUBLE_STRAND_BREAK_PROCESSING.” C–E) DNA fiber assay in OVCAR5 and OVCAR8 cells exposed to indicated treatments. C) Cells were treated with 20 µm IdU for 30 min, followed by 200 µm CIdU and indicated treatments. Representative images of DNA fibers (D) and quantifications of CIdU/ldU ratio (E) were shown. F,G) Representative images (F) and quantifications (G) of neutral comet assay in OVCAR5 and OVCAR8 cells following the indicated treatments. Scale bar, 20 µm. H,I) Representative images (H) and quantifications (I) of γH2AX foci in OVCAR5 and OVCAR8 cells following the indicated treatments. Scale bar, 2.5 µm. J) Immunoblot analysis of indicated proteins including γH2AX, Histone H3, total and phospho MCM2, and ACTIN in lysates collected from OVCAR5 and OVCAR8 cells following the indicated treatments. Data in (E,G,I) are shown as mean ± SD of triplicate (one‐way ANOVA). ^**^ *p* < 0.01; ^***^ *p* < 0.001.

To unravel the molecular mechanism underlying the synergistic effect of CDC7i and Olaparib, we conducted RNA‐sequencing (RNA‐seq) in OVCAR5 cells treated with Olaparib in the presence or absence of XL413. Gene expression profiling analysis identified 733 up‐regulated genes and 569 down‐regulated genes in the combination groups (*p* ≤ 0.05) compared to the single treatment or control groups (**Figure** **3**A). Notably, GSEA analysis unveiled significant enrichment of DNA replication and DNA double‐strand break processing signaling pathways (Figure 3B), indicating that the combined effect of XL413 and Olaparib is correlated with genome instability and replication stress. These findings are consistent with previous studies that replication fork protection and replication stress could modulate the sensitivity of PARPi.^[^ ^8^, ^22^ ^]^ In addition, using gene expression and drug response data from the GDSC and Cancer Cell Line Encyclopedia (CCLE) datasets, we performed integrated analyses with single sample gene set enrichment analysis (ssGSEA) method.^[^ ^24^, ^25^ ^]^ The results revealed a significant correlation between the abundance of the replication stress signature and the sensitivity to three different PARPi (Figure S3A–C, Supporting Information). A previous study demonstrated that CDC7 inhibition significantly reduced the initiation of DNA replication throughout S phase, leading to subsequent replication stress,^[^ ^26^ ^]^ which prompted us to investigate whether XL413 in combination with Olaparib could induce greater DNA replication stress to inhibit cell proliferation. We next conducted a DNA fiber assay to examine the progression of DNA replication fork.^[^ ^27^ ^]^ The results showed that the ratio of CldU/ldU was significantly decreased after combination treatment (Figure 3C–E), indicating a slower speed of DNA replication fork progression. In addition, we detected a significant induction of DNA double‐strand breaks upon combination treatment using the neutral comet tail assay (Figure 3F,G). Through confocal immunofluorescence, we observed a substantial increase in the number of γH2AX foci after treatment with the combination of Olaparib and XL413 in both OVCAR5 and OVCAR8 cells (Figure 3H,I), which indicated heightened DNA damage. The protein level of γH2AX was also elevated after the combination treatment (Figure 3J). Taken together, these results indicated that the combination of CDC7 inhibition and Olaparib may enhance the DNA damage and replication stress, thereby elevating the efficacy of Olaparib as a therapeutic regimen.

### 2.4 Induction of Cell‐Autonomous cGAS/STING Response by Olaparib and CDC7 Inhibitor

Genome instability and DNA replication stress are recognized as primary sources of cytosolic dsDNA, which subsequently triggers cGAS/STING pathway.^[^ ^28^ ^]^ Recent reports have suggested that PARPi might contribute to the activation of cGAS/STING.^[^ ^11^, ^12^, ^29^ ^]^ Based on these findings, we next determined whether the combination of XL413 with Olaparib could trigger cGAS/STING signaling activity, subsequently leading to the activation of type I interferon innate immune responses.

**Figure 4:**
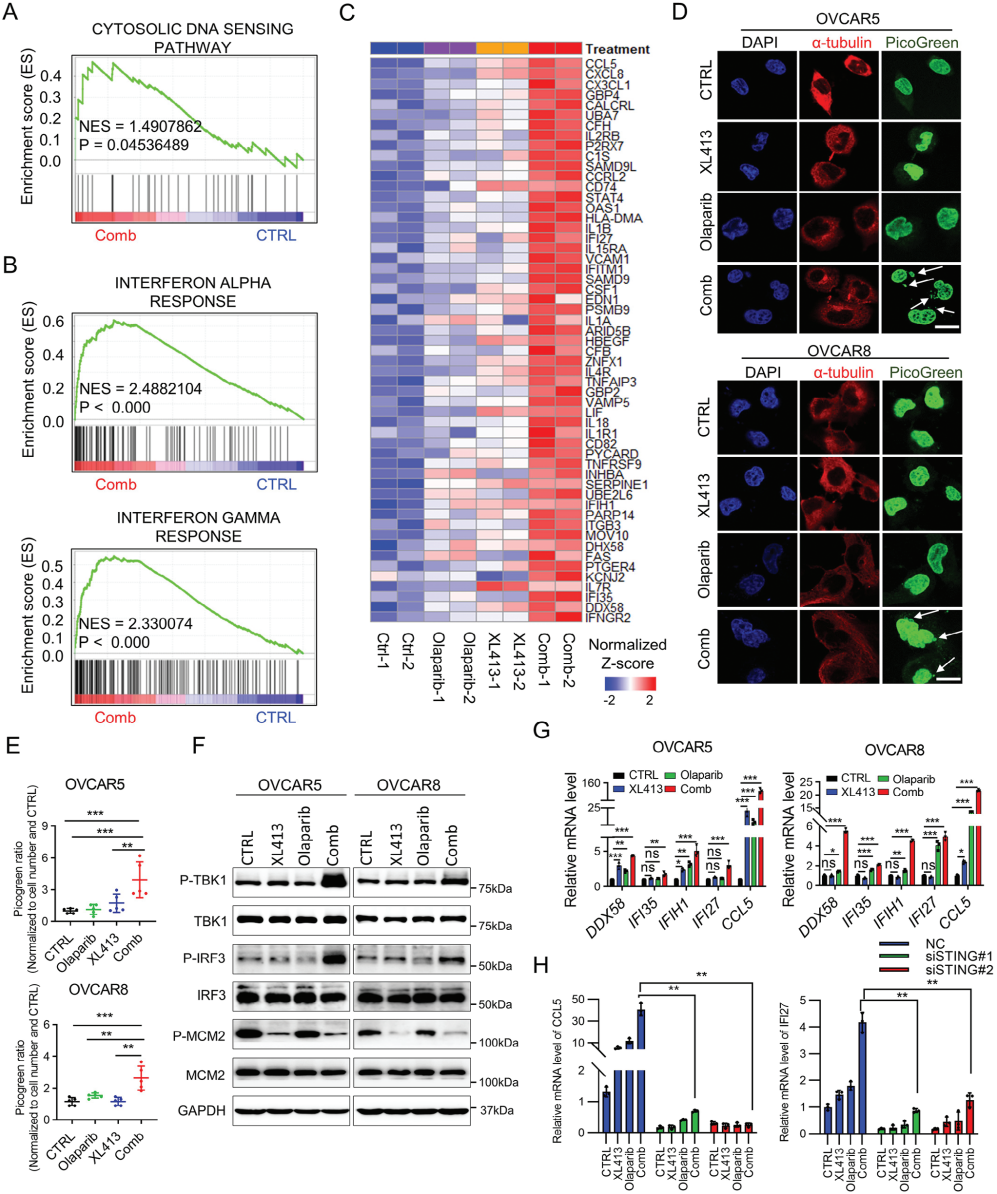
Induction of cell‐autonomous cGAS/STING response by Olaparib and CDC7 inhibitor. A,B) GSEA analysis showing the “CYTOSOLIC DNA SENSING PATHWAY” (A) and “INTERFERON ALPHA RESPONSE/INTERFERON GAMMA RESPONSE” (B) were enriched in combination treatment group using KEGG and Hallmarks gene sets. C) Heatmap of the significantly upregulated ISGs (*p* < 0.05) upon combinational treatment of XL413 and Olaparib versus control in OVCAR5 cells (Duplicates). D,E) Representative images (D) and quantifications (E) of PicoGreen staining in OVCAR5 and OVCAR8 cells following the indicated treatments. DAPI (blue) was used to visualize the nuclei. Scale bar, 5 µm. Data are shown as mean ± SD (one‐way ANOVA). ^**^ *p* < 0.01, ^***^ *p* < 0.001. F) Immunoblot analysis of indicated proteins including total and phospho TBK1, total and phospho IRF3, total and phospho MCM2, and GAPDH in lysates collected from OVCAR5 and OVCAR8 cells following the indicated treatments. G) qRT‐PCR analysis of the indicated ISGs expression levels in OVCAR5 and OVCAR8 cells after treated with CTRL, XL413, Olaparib, and combination treatment. Data are shown as mean ± SD of triplicate (one‐way ANOVA). ns, no significance, ^*^ *p* < 0.05, ^**^ *p* < 0.01; ^***^ *p* < 0.001. H) qRT‐PCR analysis of the indicated ISGs expression levels in OVCAR5 cells treated with siNC, siSTING#1 or siSTING#2. Data are shown as mean ± SD of triplicate (two‐way ANOVA). ns, no significance, ^*^ *p* < 0.05, ^**^ *p* < 0.01; ^***^ *p* < 0.001.

Using GSEA analysis on the RNA‐seq data, we observed remarkably elevated expression of the cytosolic DNA sensing pathway, which is associated with type‐I interferon responses, in OVCAR5 cells treated with the combination of drugs compared to single treatment or control group (**Figure** **4**A). In addition, the pathways related to interferon‐alpha, interferon‐gamma, and inflammatory responses were significantly upregulated, whereas E2F targets, MYC targets, and DNA repair signaling were suppressed upon combination treatment (Figure 4B; Figure S4A,B, Supporting Information). The expression levels of interferon signature genes were notably increased in the combination group (Figure 4C). Moreover, the accumulation of cytosolic DNA, recognized as a source of cGAS/STING activation,^[^ ^30^ ^]^ was found to be higher in cells treated with the combined drugs (Figure 4D,E). Consistently, the combination of Olaparib and XL413 led to increased phosphorylation of TANK‐binding kinase 1 (TBK1) and interferon regulatory factor 3 (IRF3) (Figure 4F), indicating the induction of type I interferon response. Consequently, the combination treatment resulted in the activation of interferon‐stimulated genes (ISGs) (Figure 4G). To further determine whether activation of cGAS/STING pathway mediates the effect of Olaparib and XL413, we conducted siRNA‐mediated STING knockdown in OVCAR5 cells (Figure S4C, Supporting Information), the knockdown of STING could significantly diminish the expressions of ISGs induced by the combination treatment (Figure 4H). These results indicated that the cGAS/STING pathway plays a prominent role in the immunomodulatory potential of the combination of CDC7i and Olaparib in OV cells. Taken together, the induction of the cGAS/STING response by the combined drugs highlights a potential avenue for enhancing the innate immune response within cancer cells. The activation of this cell‐autonomous immune pathway may have implications for the therapeutic efficacy of Olaparib and CDC7 inhibition in the treatment of OV.

### 2.5 Targeting CDC7 with Olaparib Triggers Anti‐Tumor Immunity In Vitro

**Figure 5:**
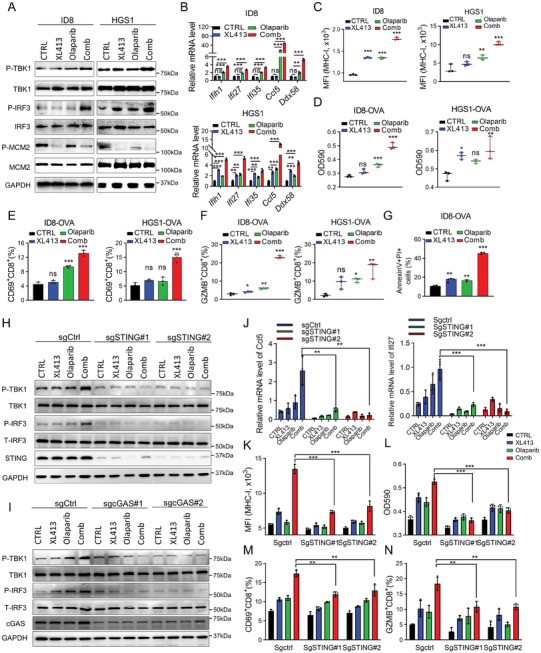
Targeting CDC7 with Olaparib triggers anti‐tumor immunity in vitro. A) Immunoblot analysis of indicated proteins including total and phospho TBK1, total and phospho IRF3, total and phospho MCM2, and GAPDH in lysates collected from ID8 and HGS1 cells following the indicated treatments. B) qRT‐PCR analysis of the indicated ISGs expression levels in ID8 and HGS1 cells after the indicated treatments. C) Expression levels of MHC‐I in ID8 and HGS1 cells after the indicated treatments. D) ID8‐OVA or HGS1‐OVA cells were treated with the indicated treatments, and then co‐cultured with B3Z cells, B3Z activation was determined by LacZ activity. E,F) Pretreatment of ID8‐OVA or HGS1‐OVA cells with the indicated treatments, and then co‐cultured with OT‐I cells, OT‐I activation was determined by expression of CD69 (E) and GZMB (F) by flow cytometry analysis. G) The cytotoxic effect of OT‐I of ID8‐OVA cells was detected after co‐culturing with OT‐I cells for 48 h. H,I) Immunoblot analysis of indicated proteins including total and phosphor‐TBK1, total and phosphor‐IRF3, STING, and GAPDH in lysates collected from HGS1 cells transfected with sgCtrl or sgSTING (‐1, ‐2) or sgcGAS (‐1, ‐2) following the indicated treatments. J) qRT‐PCR analysis of Ccl5 and Ifi27 in HGS1‐OVA WT or STING^−/−^ cells after the indicated treatments. K) Surface levels of MHC‐I in HGS1‐OVA WT or STING^−/−^ cells after treated with XL413 or Olaparib or their combination for 48 h. L) HGS1‐OVA WT or STING^−/−^ cells were treated with XL413 or Olaparib or their combination for 48 h, then co‐cultured with B3Z for an additional 24 h, after which B3Z activation was determined by LacZ activity. M,N) HGS1‐OVA WT or STING^−/−^ cells were treated with XL413 or Olaparib or their combination, then co‐cultured with OT‐I, after which OT‐I activation was determined by expression of CD69 (M) and GZMB (N) by flow cytometry analysis. Data in (B–G) are shown as mean ± SD of triplicate (one‐way ANOVA). Data in (J–N) are shown as mean ± SD of triplicate (two‐way ANOVA). ns, no significance, ^*^ *p* < 0.05, ^**^ *p* < 0.01, ^***^ *p* < 0.001.

Given the correlation between activation of cGAS/STING signaling and type I interferon innate immune responses, we next sought to assess the effects of Olaparib in combination with CDC7 inhibition on anti‐tumor immunity in vitro. We utilized two murine OV cells, ID8 and HGS1. The data showed that the combination of Olaparib with XL413 significantly inhibit cell proliferation (Figure S5A, Supporting Information), with concomitant increases in cytoplasmic DNA levels (Figure S5B, Supporting Information). Consistent with our findings in human OV cells, the combination treatment resulted in increased levels of phosphorylated TBK1 and IRF3 (**Figure** **5**A), along with the induction of ISGs (Figure 5B). Notably, major histocompatibility complex Class I (MHC‐I) expression, which is crucial for tumor antigen presentation and T cell activation, was enhanced on the tumor cell surface upon combination treatment (Figure 5C). We also introduced OVA cDNA into ID8 and HGS1 cells and observed a significantly increased expression level of MHC‐I bound SIINFEKL complex after the combination treatment compared to single treatment or control (Figure S5C, Supporting Information). These findings indicated that CDC7i, in combination with Olaparib, can stimulate anti‐tumor immune responses through promoting antigen presentation and activating the cGAS/STING pathway, resulting in interferon‐induced immune response.

GSEA analysis showed markedly upregulated expression of genes associated with T cell activation in cells treated with Olaparib and XL413 compared with control (Figure S5D, Supporting Information). In addition, recent studies reported that the anti‐tumor immunity effects of PARPi were mediated by CD8+ T cell activation.^[^ ^11^, ^31^, ^32^ ^]^ Based on these findings, we next determined whether the combination of XL413 with Olaparib could potentiate the activation of CD8+ T cells, we utilized a LacZ assay to assess the activation of OVA‐specific CD8+ T cell hybridoma B3Z cells co‐cultured with ID8‐OVA or HGS1‐OVA cells.^[^ ^33^ ^]^ The results showed that the combination treatment significantly enhanced LacZ activity (Figure 5D), a reporter for indicating T‐cell activation. In addition, when cells were co‐cultured with primary OT‐I T cells, the levels of CD8+ T cells expressing the activation marker CD69 and the effector molecules granzyme B (GZMB) and IFNγ were also elevated after combination treatment (Figure 5E,F; Figure S5E, Supporting Information). Furthermore, we investigated the cytotoxic killing effect of OT‐I cells on ID8‐OVA cells using the annexin V/propidium iodide assay and the lactate dehydrogenase (LDH) release assay. The data showed that the combination treatment significantly increased the proportion of apoptotic cells and LDH release (Figure 5G; Figure S5F, Supporting Information). To further verify that the combination treatment promoted immune activation through cGAS/STING pathway, we performed CRISPR/Cas9 mediate‐knockout targeting STING or cGAS in HGS1‐OVA cells with two individual sgRNAs (Figure S5G, Supporting Information) and found that either the absence of cGAS or STING significantly suppressed the elevation of phosphorylation of IRF3 and TBK1 and the increased expression of ISGs and MHC‐I expression induced by the combination treatment (Figure 5H–K; Figure S5H,I, Supporting Information). More importantly, the induced LacZ activity and levels of CD8+ T cells expressing GZMB and CD69 were markedly attenuated after cGAS or STING knockout (Figure 5L–N; Figure S5J, L, Supporting Information). These findings collectively demonstrated that targeting CDC7 with Olaparib trigger anti‐tumor immunity through activating cGAS/STING signaling pathway and boosts T cell activation, suggesting its potential as an effective therapeutic strategy.

### 2.6 CDC7 Inhibition Significantly Augments Anti‐Tumor effect of Olaparib In Vivo

**Figure 6:**
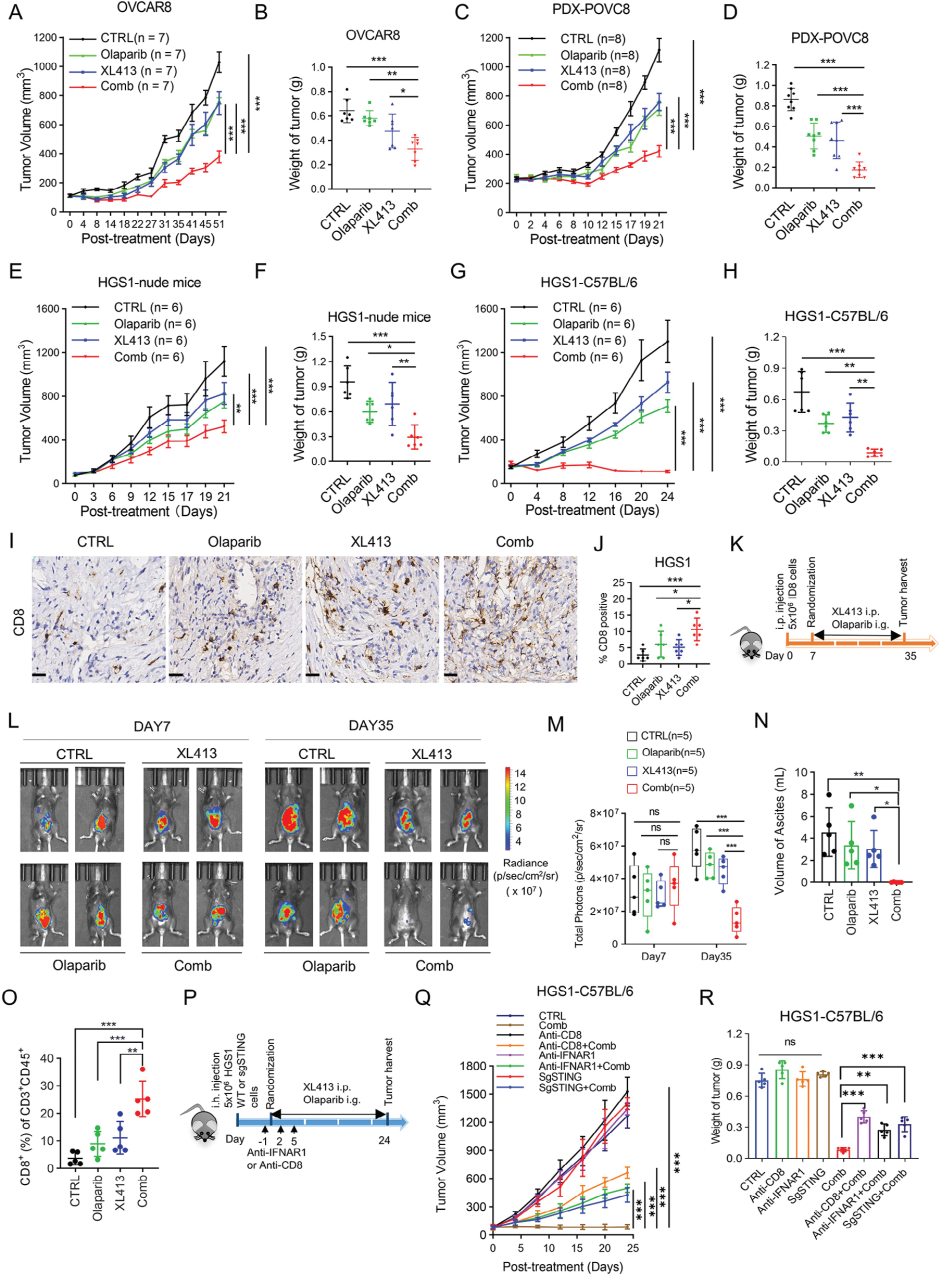
CDC7 inhibition significantly augments anti‐tumor effect of Olaparib in vivo. A,B) Tumor growth curve (A) and tumor weight (B) of nude mice injected with OVCAR8 cells. Mice were treated with CTRL, Olaparib, XL413, or combination therapy (n = 7). C,D) Tumor growth curve (C) and tumor weight (D) of PDX model (PDX‐POVC8). Mice were treated with CTRL, Olaparib, XL413, or combination therapy (n = 8). E,F) Tumor growth curve (E) and tumor weight (F) of nude mice injected with mouse OV HGS1 cells. Mice were treated with CTRL, Olaparib, XL413, or combination therapy (n = 6). G,H) Tumor growth curve (G) and tumor weight (H) of C57BL/6 mice injected with mouse OV HGS1 cells. Mice were treated with CTRL, Olaparib, XL413, or combination therapy (n = 6). I,J) Representative images (I) and quantifications (J) of CD8 immunohistochemistry in mice bearing HGS1 cells of experiments described in Figure 6G. Scale bar, 25 µm. K–O) C57BL/6 mice were injected intraperitoneally with ID8 cells. Seven days after transplantation, tumor‐bearing mice were randomized into the indicated treatment groups (n = 5). After 4 weeks treatment, mice were euthanized and their tumors were harvested for further analysis. K) Scheme of the experimental design. L) Representative bioluminescence images of mice bearing ID8 cells at day 7 and day 35. M) The bar graph showed the change in bioluminescence in mice. N) Ascites produced in the indicated treatment groups were quantified at the end of treatment. O) Percentages of tumor‐infiltrated CD8+ T cells in the indicated treatment were assessed by flow cytometry analysis. P) Experimental design to evaluate combination effect of XL413 and Olaparib after STING‐KO, blockade of CD8 or IFNAR1 in mice bearing HGS1 cells. Q) Tumor growth curves ± SD from CTRL, XL413 plus Olaparib (Comb), anti‐CD8 alone, Comb + anti‐CD8, sgSTING alone, Comb + sgSTING, anti‐IFNAR1 alone, and Comb + anti‐IFNAR1 treatment groups. R) Tumor weight in HGS1 mice described in Figure 6Q (n = 5). Data in (A,C,E,G,M,Q,R) are shown as mean ± SD (two‐way ANOVA). Data in (B,D,F,H,J,N,O) are shown as mean ± SD (one‐way ANOVA). ns, no significance, ^*^ *p* < 0.05, ^**^ *p* < 0.01, ^***^ *p* < 0.001.

To investigate the combination effect Olaparib and XL413 in vivo, we assessed the efficacy and toxicity of this potential therapeutic combination in OVCAR8 xenograft model and a patient‐derived xenograft (PDX) model (PDX‐POVC8).^[^ ^34^ ^]^ The results showed that the combination of XL413 and Olaparib significantly inhibited tumor growth compared to XL413 or Olaparib alone with a modest change in body weight (**Figure** **6**A–D; Figure S6A,B, Supporting Information). Given that Olaparib combined with CDC7i triggers anti‐tumor immunity in vitro, we inoculated HGS1 cells in T cell‐deficient nude mice and syngeneic immunocompetent C57BL/6 mice. The combination treatment effectively suppressed tumor growth in nude mice bearing HGS1 tumors (Figure 6E,F), while achieving complete tumor regression in the immunocompetent C57BL/6 mouse model, indicating host immune involvement in combination treatment response (Figure 6G,H). Minimal changes in body weight of mice in all three treatment groups compared to control revealed minimal treatment toxicity (Figure S6C,D, Supporting Information), which is further confirmed by hematoxylin and eosin staining of various organs and liver and kidney function (Figure S6E,F, Supporting Information). To assess the impact of XL413 plus Olaparib treatment on the tumor microenvironment, we analyzed the tumor‐infiltrating immune cells and found that the combination treatment significantly increased the percentage of tumor‐infiltrating CD8+ T cells, but not that of CD4+ T cells or B cells (Figure S6G, Supporting Information). In addition, no significant difference was detected in dendritic cell (DCs), macrophages, and natural killer (NK) cells (Figure S6H, Supporting Information). The results are further confirmed with immunohistochemistry (IHC) analysis that showed a significant increase in the tumor infiltration of CD8+ T cells following the combination treatment (Figure 6I,J). Consistently, a higher proportion of tumor‐infiltrating T cells exhibited expression of the T cell activation marker CD69 and effector molecules GZMB and IFNγ (Figure S6I, Supporting Information) in the combination treatment group as compared with to the control or single drug treatment group. Furthermore, we also examined the anti‐tumor immunity of Olaparib and XL413 in immunocompetent mouse model injected with ID8 cells intraperitoneally (Figure 6K). Notably, the combination treatment was significantly more effective in reducing tumor burden and ascites production and a modest change in body weight compared with either of the single treatments (Figure 6L–N; Figure S6J, Supporting Information). We also detected a remarkably higher proportion of tumor‐infiltrating CD8+ T cells in the combination group using flow cytometry analysis (Figure 6O). To validate the function of cGAS‐STING‐type I interferon pathway and CD8+ T cells in the combination therapy in vivo, we then carried out an additional experiment by treating tumor‐bearing mice with IFNAR1 or CD8 blocking antibodies, the efficacy of the combination therapy was significantly impaired by IFNAR1 or CD8 neutralization. More importantly, knocking out of STING also substantially abolished the synergistic effect of XL413 with Olaparib (Figure 6P–R; Figure S6K,L, Supporting Information). Consistently, the induced tumor‐infiltrating CD8+ T cells by the combination treatment group relative to the CTRL group were mitigated by IFNAR1 or CD8 neutralization or STING knockout (Figure S6M,N, Supporting Information). Altogether, these observations indicated that the combination of Olaparib and XL413 inhibits tumor growth and exerts anti‐tumor immunity through cGAS‐STING‐type I interferon pathway and potentiating the function CD8+ T cells in OV. The enhanced anti‐tumor effects of Olaparib in combination with CDC7 inhibition hold promise for the development of more effective therapeutic strategies against OV.

**Figure 7:**
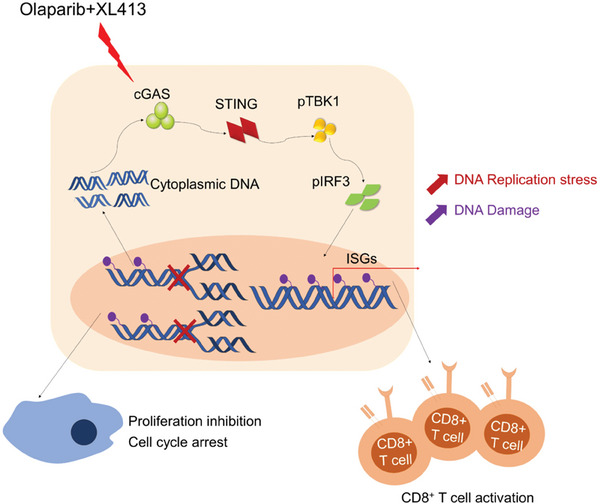
Schematic model illustrating the anti‐tumor efficacy of combination treatment of CDC7 inhibitor and Olaparib in OV. Combined CDC7 inhibition and Olaparib promote genome instability and enhance DNA replication stress, which significantly upregulates ISGs expression through cGAS/STING activation to boost antitumor efficacy in vitro and in vivo.

## 3 Discussion

BRCAs are essential for the repair process of DNA damage caused by DNA lesions such as DNA replication forks stalling or DNA double‐strand breaks (DSBs) by HRR.^[^ ^35^ ^]^ Although PARPi was initially designed for patients with BRCA1/2 mutations, our study found no significant correlation between Olaparib sensitivity and the mutation status of DNA damage response genes or HRD scores in OV cell lines. Further in vitro studies, including those utilizing BRCA1/2 deficient and proficient cells as well as PARPi acquired resistant cells, supported the lack of correlation between Olaparib sensitivity and BRCA mutation or HRR status. This is consistent with the mounting volume of clinical and mechanistic studies highlighting the therapeutic responses of PARPi in a wider cohort of OV patients irrespective of BRCA1/2 status or HR‐mediated DNA repair deficiency.^[^ ^36^, ^37^ ^]^ Our findings have helped to address the existing gap in understanding the targets correlated with sensitivity to PARPi in OV. Identifying such targets reveals vulnerabilities that can be leveraged to enhance the anti‐cancer effects of PARPi, particularly given that resistance to PARPi often develops over time.

By overlapping CRISPR screens for Olaparib regulators^[^ ^20^ ^]^ with over‐expressed genes in OV, we identified that 35 genes were upregulated in OV and associated with sensitivity to PARPi. Pathway enrichment analysis revealed a significant enrichment of cell cycle‐related pathways, which were further validated in another cohort that contained datasets on PARPi‐sensitive and acquired resistant cells. These results illustrated that Olaparib sensitivity is associated with aberrant activation of cell cycle‐related pathways. Cell cycle signaling is a well‐known regulator of tumor development and drug resistance.^[^ ^38^ ^]^ Targeting these pathways has been shown to improve clinical outcomes, including prolonged survival and enhanced therapeutic responses,^[^ ^39^, ^40^, ^41^ ^]^ even for cancers that are resistant to PARPi.^[^ ^42^, ^43^, ^44^, ^45^, ^46^ ^]^ Specifically, our drug screening identified CDC7, a protein crucial for the G1/S phase transition and DNA replication, as a potential target. Over‐expression of CDC7 has been reported to be associated with poor prognosis in a range of cancers,^[^ ^47^, ^48^, ^49^, ^50^ ^]^ including OV. Several studies have reported that inhibition of CDC7 exhibits potent anti‐tumor activity and promotes drug sensitivity through multiple mechanisms in preclinical models,^[^ ^51^, ^52^, ^53^ ^]^ some of which have progressed into clinical trials for cancer treatments.^[^ ^54^ ^]^ Further knockdown of CDC7 in resistant cells synergized with PARPi. We extensively validated the combination effect of CDC7i and PARPi both in vitro and in vivo, especially with patient‐derived cells and xenograft models, providing strong preclinical evidence of its enhanced efficacy and low toxicity. These findings suggest that combining CDC7i with PARPi could be a promising strategy for OV patients who have failed single‐agent Olaparib therapy and warrant further investigation in clinical trials. Further studies are required to explore if other pathways, other than cell cycle pathway, could affect PARPi sensitivity.

Currently, immunotherapy has shown promising results in multiple cancers, but exhibits limited response in OV.^[^ ^55^, ^56^ ^]^ The highly immunosuppressive tumor microenvironment of OV is associated with limited efficiency in immunotherapy.^[^ ^57^, ^58^ ^]^ The cGAS/STING pathway plays a significant role in the activation of the interferon response and the induction of antitumor immunity.^[^ ^59^ ^]^ Genome instability and DNA replication stress are primary sources of micronuclei formation and the generation of cytosolic DNA, which subsequently triggers the activation of the cGAS/STING pathway.^[^ ^28^ ^]^ Activation of cGAS/STING through DNA damage and replication stress induced by PARPi has been recognized as an important component of their synthetic lethal effects.^[^ ^60^, ^61^ ^]^ PARPi have been reported to induce immune‐activation and have good therapeutic effects when combined with immunotherapy.^[^ ^12^, ^32^ ^]^ Here, our study revealed that the combination of Olaparib and XL413 resulted in significantly higher cell cycle arrest and stalled replication forks and significant induction of DNA double‐stranded breaks in comparison to Olaparib, thus inducing a significant formation of cytoplasmic DNA, which finally trigger cGAS/STING signaling. Our in vivo study indicated that the therapeutic effects of the combination of Olaparib with XL413 were greater in immunocompetent mice models, as compared to immunodeficient mice models, suggesting the implication of the immune system in enhancing the anti‐cancer responses. Further investigation of tumor infiltration of immune cells and cGAS/STING knockout studies identified CD8+ T‐cell recruitment via STING pathway activation as a critical determinant of the anti‐tumor immunity efficacy of PARP inhibition and CDC7i in OV (**Figure** **7**). These findings provide substantial preclinical evidence to support the integration of immunotherapy into treatment regimens for OV.

In summary, our study identified a highly effective therapeutic strategy by combining CDC7i with PARPi in OV. This combination not only enhances anti‐cancer responses but also leverages the immune system to improve therapeutic outcomes. Overall, our study provides robust preclinical data that supports the need for further clinical trials to evaluate the efficacy and safety of the CDC7i and PARPi combination. This research paves the way for optimizing therapeutic strategies, thereby enhancing the potential for successful outcomes in OV treatment.

Despite the promising findings, our study has several limitations. While our study showed that CDC7i enhance the effects of PARPi, the mechanisms by which CDC7i amplify DNA replication stress and genomic instability are not yet fully understood, additional studies are needed to better understand the underling molecular mechanisms. Additionally, we found that either the absence of cGAS or STING significantly suppressed immune activation and anti‐tumor effect induced by the combination treatment. These observations raise the question of whether the combination therapy would be relatively more effective in patients with high cGAS/STING expression, which is warranted for further exploration to identify patients who are more suitable for the combination therapy, thus further improving the clinical value of PARPi. Furthermore, Further investigation is needed to assess the impact of this combination, especially when combined with immunotherapy, across different BRCA/HRR gene mutation backgrounds in clinical or preclinical models.

## 4 Experimental Section

### Cell Lines and Reagents

Human OV commercial cell lines COV504 and A2780 were obtained from the European Collection of Cell Cultures (Salisbury, UK). Human OV commercial cell lines KURAMOCHI and OVCAR8 were obtained from the Japanese Collection of Research Bioresources Cell Bank (Tokyo, Japan) and the National Cancer Institute (USA). All other human OV commercial cell lines were purchased from the American Type Culture Collection (ATCC). All cells have been authenticated by Short Tandem Repeat (STR) sequencing. POVC cells POVC8 and POVC17 were produced in our previous study.^[^ ^34^ ^]^ Mouse OV cell line ID8 was purchased from the Cell Bank of the Chinese Academy of Sciences (Shanghai, China). Mouse OV cell line HGS1 was a gift from Prof. Balkwill (Barts Cancer Institute).^[^ ^62^ ^]^ The B3Z hybridoma T cells were gifts from Nilabh Shastri (University of California, Berkeley, California, USA).^[^ ^33^ ^]^ All cells were maintained at 37 °C in a humidified 5% CO_2_ atmosphere with DMEM or RPMI 1640 medium containing 10% FBS (HyClone) and 1% penicillin‐streptomycin (Gibco). No mycoplasma contamination was detected in all cells. Olaparib was purchased from Selleck Chemicals, while XL413, PHA‐767491, Niraparib, and Talazoparib were purchased from Targetmol (Boston, MA).

### Generation of Acquired Resistant Cell Line to Olaparib

Generation of OVCAR3 cell line resistant to Olaparib was performed as per our previous report.^[^ ^34^ ^]^ Briefly, OVCAR3 cells were grown with increasing concentrations of Olaparib. Living cells were exposed to Olaparib at the same dose for three‐passages periods. Acquired resistant cells were maintained in the presence of Olaparib and validated with multiple assays.

### Colony Formation Assay

1–2 × 10^4^ cells were plated in six‐well plates and maintained for 10–12 days following different treatments. Colonies were fixed with methanol, stained with gentian violet, and photographed by ChemiDoc Imagers (Bio‐Rad).

### Cell Proliferation Assay

1–2 × 10^3^ cells were seeded in a 96‐well plate in triplicate for 20 h. The cells were treated with different drugs or experimental treatments for 96 h. Cell proliferation rates were measured using CellTiter‐Glo Luminescent Cell Viability Assay (Promega) following the manufacturer's protocols.

### Drug Screening

A 130 customized cell cycle‐related compounds library (TopScience, Table S1, Supporting Information) was used for drug screening combined with Olaparib (2.5 um). 2 × 10^3^ OVCAR5 or OVCAR8 cells were seeded in a 96‐well plate and treated with 130 compounds alone or combined with Olaparib for 96 h. Cell proliferation rates were measured by CellTiter‐Glo Luminescent Cell Viability Assay (Promega) following the manufacturer's protocols. The S/C score (Single/Combination) was calculated to determine the differential drug sensitivity.

### Immunoblot Analysis

Protein extracts were prepared with RIPA cell lysis buffer using the protease inhibitor cocktail (Roche, Basel, Switzerland). Protein concentrations were measured using Pierce BCA protein assay (Thermo Fisher Scientific, Waltham, MA). Lysates and prestained protein markers (M221, GenStar) were subjected to SDS‐PAGE and transferred to PVDF membrane for immunoblot analysis as described previously.^[^ ^63^ ^]^ The following antibodies were used for immunoblot analysis: CDC7 (Santacruz, sc‐56275), GAPDH (Proteintech, 60004‐1‐1 g), ACTIN(CST, 8456S), γH2AX (CST, 2577S), H3 (CST, 3638S), P‐MCM2 (Abcam, ab133243), MCM2 (Abcam, ab108935), P‐TBK1 (CST, 5483S), TBK1 (CST, 3504S), P‐IRF3 (CST, 4947S), IRF3 (CST, 4302S), cGAS (CST, 31659), STING (CST, 13467), BRCA1 (CST, 9010) and a‐tublin (Proteintech, 66031‐I‐1 g). The secondary antibodies were HRP‐conjugated anti‐rabbit and anti‐mouse (GE Healthcare Life Sciences NA934 and NA931). Membranes were exposed using ECL Western Blotting Substrate (MK‐S400, MIKX, Shenzhen, China).

### IHC Analysis

The FFPE sections were dewaxed and rehydrated through graded alcohol to water prior to antigen unmasking by heat retrieval in pH 6.0 Target Retrieval Solution for 20 min (Dako Cytomation, Denmark). After treatment with 3% hydrogen peroxide, the sections were incubated with optimally diluted antibody targeting mouse CD8 antibody (CST, 98 941) for 60 min at room temperature. Detection was carried out using Dako REAL HRP Rabbit detection kit for 30 min. DAB staining was performed according to manufacturer's instructions (Dako Cytomation). The sections were counterstained with Gill's Hematoxylin, dehydrated, cleared, and mounted in Canada Balsam mounting medium. The stained sections were scored for intensity of staining in the cytoplasmic and nuclear compartments.

### RNA Interference

Cells were plated into six‐well plates and transfected with indicated siRNAs using Lipofectamine RNAi‐MAX (LifeTechnologies) following the protocols. The medium containing siRNAs and Lipofectamine were changed with a fresh complete medium after 6 h of transfection. Cells were maintained for 20 h and digested for other experiments. All sequences of siRNAs used in this project are available in Table S2 (Supporting Information).

### Confocal Immunofluorescence Analysis

Cells were fixed with 3.7% paraformaldehyde, permeabilized with 0.2% Triton X‐100 and blocked with 1% BSA for 1 h at room temperature. The indicated primary antibodies were diluted for incubation overnight at 4 °C. After washed with PBS, incubated with Alexa Fluor 594–conjugated rabbit and Alexa Fluor 647–conjugated mouse secondary antibodies (Thermo Fisher Scientific) in IFF for 1 h at 4 °C, cells were incubated with DAPI (MIKX, CE378) with/without 1:400 PicoGreen (Ouant‐iT Pico‐Green dsDNA reagent, Thermo Fisher Scientific) for 15 min at room temperature. Subsequent labeling, imaging, and image analysis steps were performed as described previously.^[^ ^33^ ^]^ The following primary antibodies were used for immunoblot analysis: a‐tubulin (Proteintech, 66031‐I‐1 g, 1:500), γH2AX (CST, 2577s, 1:200).

### Plasmids Construction and Virus Infection

The plasmids targeting mouse STING and cGAS were designed using the Optimized CRISPR Design (http://chopchop.cbu.uib.no/) and cloned into the LentiCRISPR v2 vector (Addgene plasmid 52 961) containing the Streptococcus pyogenes Cas9 nuclease gene. For human BRCA1 gene knockout, two sgRNA sequences were obtained as referenced by^[^ ^64^, ^65^ ^]^ and a third sgRNA was designed using the Optimized CRISPR Design (http://chopchop.cbu.uib.no/) and sgRNAs were then inserted into the LentiCRISPR v2 vector. Human BRCA1 was cloned into the pCDH‐CMV lentiviral expression vector (System Biosciences) and shRNA targeting CDC7 was inserted into the PLKO.1 lentiviral vector. The lentiviral vectors were co‐transfected with packaging plasmids pspax2 and pMD2.G into 293T cells. At 48 h post‐transfection, the viral supernatants were harvested and added to the target cells for another 48 h. Puromycin was used for selection of positive cells. All sequences are listed in Table S2 (Supporting Information).

### qRT‐PCR and RNA‐Seq

Total RNA was obtained from cells using RNeasy Mini Kit (QIAGEN) or EZ‐press RNA purification kit (EZBioscience) and remove DNA with DNase I (GMP‐E127, Novoprotein) according to the manufacturer's protocol. qRT‐PCR was performed using a reverse transcription kit (AT341‐02, Transgen Biotech) and then a quantitative kit (N30920, Transgen Biotech) on a Bio‐Rad CFX Real‐Time PCR machine. For RNA‐Seq, the libraries were prepared as reported previously.^[^ ^34^ ^]^ All sequences of qRT‐PCR primers are available in Table S2 (Supporting Information).

### Cell Cycle Analysis and Apoptosis Assay

Cells were treated with indicated agents for 72 h. For cell cycle analysis, cells were harvested and fixed with 70% ethanol. Fixed cells were stained with propidium iodide and Alexa Fluor 647 conjugated p‐H3 (S28) according to procedures described in previous publications.^[^ ^63^ ^]^ For apoptosis assay analysis, Apoptotic cells were quantified using the Annexin V–FITC Apoptosis Detection Kit (Vazyme). Data were analyzed using BD LSRFortessa X‐20 (BD Biosciences) and the flow cytometry Spectral Cell Analyzer SP6800Z (Sony Biotechnology, Tokyo, Japan).

### Comet assay

Cells were harvested and washed with cold PBS twice, and the cells were diluted at a concentration of 1 × 10^5^ cells mL^−1^ in PBS. Briefly, 50 µL of cells (1 × 10^5^ cells mL^−1^) was mixed in 500 µL of 1% low‐melting agarose and the mixture was added onto comet slides. The coated slides were incubated in lysis solution (2.5 m NaCl, 100 mm Na_2_EDTA, 10 mm Tris‐base, 10% DMSO, 1% Triton X‐100, pH 10) for 2 h at 4 °C. The slides were treated with alkaline solution (300 mm NaOH, 1 mm EDTA, pH >13) for 20 min at room temperature. The electrophoresis was performed using electrophoresis solution (300 mm NaOH, 1 mm EDTA, pH>13) at 25 V, 300 mA for 30 min. The slide was stained with propidium iodide and analyzed using OpenComet software.^[^ ^66^ ^]^ The level of DNA damage was presented as percentage of DNA in tail.

### HRR Assay

HRR reporter systems containing I‐Sce I expression plasmid (pCBASce) and reporter plasmid (DR‐GFP) were a gift from Prof. Zhang (Shenzhen University), HRR assay was performed using an as previously described.^[^ ^67^ ^]^ Briefly, pCBASce and DR‐GFP were co‐transfected into OVCAR3‐P/‐R cells for 48 h, GFP+ cells were analyzed by flow cytometry Spectral Cell Analyzer SP6800Z (Sony Biotechnology, Tokyo, Japan).

### DNA Fiber Assay

Cells were first treated with 20 µm ldU for 30 min, followed by 200 µm CIdU treatment, and then exposed to indicated treatments for 30 min at 37 °C. DNA fiber assay was performed as described previously.^[^ ^68^ ^]^ Representative images of DNA fibers were photographed using a fluorescence microscope.

### Detection of Genomic DNA in Cytosolic Extracts

The detection procedures for cytosolic genomic DNA were performed using the previously described methods.^[^ ^69^ ^]^ Cytosolic DNA was extracted after being treated with different treatments and the levels of genomic DNA (Polg1) were determined by qRT‐PCR analysis with specific primers, forward primer, 5′‐GATGAATGGGCCTACCTTGA‐3′, and reverse primer, 5′‐TGGGGTCCTGTTTCTACAGC‐3′.

### LacZ Reporter Assays

The LacZ reporter assay was performed using a co‐culture system with ID8‐OVA or HGS1‐OVA cells and B3Z cells as previously described.^[^ ^33^ ^]^ In brief, the lysed and freeze‐thawed cells were mixed with a total of 150 µL/well substrate solution (50 µL PBS containing 0.5% BSA + 100 µL β‐galactosidase buffer containing 1 mg mL^−1^ chlorophenol red β‐d‐galactopyranoside) and then incubated at 37 °C for 12–18 h, and the LacZ activity was measured using an Infinite M200 plate reader (Tecan) (590 nm).

### T Cell Activation and Cytotoxic T Lymphocyte (CTL) Assay

Tumor cells were treated with different treatments for 48 h and then stained with MHC‐I (Bio‐Legend, catalog 116 525) or MHC‐I SIINFEKL (eBioscience, catalog 17‐5743‐82). The ID8‐OVA or HGS1‐OVA cells after being treated with indicated drugs for 48 h were co‐cultured with B3Z cells for 24 h and subsequently determined the LacZ activity. When tumor cells were co‐cultured with non‐activated OT‐I T cells, surface marker CD69 (BioLegend, catalog 104 514) was stained and analyzed by flow cytometry. For intracellular markers staining, GolgiStop reagent (1000×; BD Biosciences) was added to the co‐culture system for 3 h before staining of anti‐IFN‐γ (eBioscience, catalog 17‐7311‐82) or anti‐GZMB (eBioscience, catalog 12‐8898‐82) using an intracellular fixation and permeabilization buffer kit (eBioscience) following the protocols. The stained cells were then analyzed using flow cytometry. CTL assays were performed as in our previous study.^[^ ^33^ ^]^

### In Vivo Studies

Six‐ to eight‐week‐old female C57BL/6, BALB/c nude and NOD‐SCID mice were obtained from the Beijing Vital River Laboratory Animal Technology Company and housed in the Laboratory Animal Center of Sun Yat‐sen University. All animal studies were conducted in compliance with animal protocols approved by the Institutional Animal Care and Use Committee at Sun Yat‐sen University (approval number SYSU‐IACUC‐2022‐000519). For the OVCAR8 tumor xenograft experiment, OVCAR8 (5 × 10^6^) cells were subcutaneously transplanted in the left flanks of nude mice. For PDX mouse models, the previously constructed PDX‐POVC8^[^ ^34^ ^]^ tumor masses were passaged with NOD‐SCID mice. In HGS1 mouse model, HGS1 cells (5 × 10^6^) were inoculated subcutaneously into the left flanks of nude mice or C57BL/6 mice. The mice were randomly divided into 4 groups with similar tumor size when tumor volume reached 80–100 mm^3^ and then treated with CTRL, XL413 (40 mg kg^−1^, intraperitoneal injection i.p.), Olaparib (100 mg kg^−1^, oral gavage i.g.), and combination treatment every 2 days. Tumor volume and body weight of mice were monitored every 3–5 days. When tumor volume reached 1000–1500 mm^3^, the mice were euthanized by CO_2_ inhalation, and their tumors were collected for further investigation. In the HGS1‐C57BL/6 models, the terminal blood sample was collected for examination of liver and kidney function and the tumors were collected for flow cytometry analysis for tumor‐infiltrating immune cells. Histopathological examination of the major organs was performed with hematoxylin and eosin staining. For analysis of tumor‐infiltrating immune cells, comparable mouse tumors were dissected and filtered through 70 µm cell strainers to produce single‐cell suspensions. The cells were then stained with the following antibodies and analyzed using flow cytometry BD Fortessa X‐20: CD45 (BioLegend, catalog 103116), CD3‐PE (BioLegend, catalog 100308), CD3‐APC (BioLegend, catalog 100312), CD8 (BioLegend, catalog 100706), CD4 (BioLegend, catalog 116016), NK1.1 (BioLegend, catalog 108721), CD19 (BioLegend, catalog 115549), CD11b (BD Biosciences, catalog 563015), F4/80 (BioLegend, catalog 123135), CD11c (BioLegend, catalog 117318), anti‐IFN‐γ (eBioscience, catalog 25‐7311‐82), anti‐GZMB (BioLegend, catalog 396408), CD69 CD69 (BioLegend, catalog 104514). The gating strategies for analysis of tumor‐infiltrating immune cells are available in (Figure S7, Supporting Information).

For the intraperitoneal model, ID8 cells (5 × 10^6^) were injected into the peritoneal cavity of C57BL/6 mice. Seven days after transplantation, tumor‐bearing mice were randomized into the following treatment groups (n = 5), CTRL, XL413 (80 mg kg^−1^, i.p.), Olaparib (200 mg kg^−1^, i.g.), and combination treatment. Tumor progression was monitored weekly using the In Vivo Imaging System from Caliper Life Science and results were expressed in radiance (p/s/cm^2^/sr). After 4 weeks of treatment, mice were euthanized and their tumors and ascites were harvested for further analysis.

For CD8/IFNAR1 depletion and STING Knockout experiments, HGS1 or HGS1 STING^−/−^ cells (5 × 10^6^) were inoculated subcutaneously into the left flanks of C57BL/6 mice. The mice were randomly divided into eight groups with similar tumor size when tumor volume reached 80–100 mm^3^ and then treated with CTRL or the combination treatment (XL413 (40 mg kg^−1^, i.p.) + Olaparib (100 mg kg^−1^, i.g.) every 2 days. Anti‐IFNAR‐1 (200 µg per mice, BioXcell, catalog BE0241) or anti‐CD8 (100 µg per mice, BioXcell, catalog BE0004‐1) antibodies were i.p. injected into the C57BL/6 mice three times (at day −1, 2, 5) post the drug treatment. Tumor volume and body weight of mice were monitored every 3–5 days. When tumor volume reached 1000–1500 mm^3^, the mice were euthanized by CO_2_ inhalation, and their tumors were collected for further investigation.

### Bioinformatics Analyses

Raw reads were filtered and cleaned by fastp software (version 0.12.5) with default settings and then mapped to human reference genome (GRCh38, hg38) with STAR (version 2.7.0f).^[^ ^70^, ^71^ ^]^ Accurate transcript quantification from RNA‐Seq data was calculated using RSEM software with default settings.^[^ ^72^ ^]^ The differentially expressed genes (|log2foldchange| > 1 and *p*‐value < 0.05) were estimated using DESeq2 package and pathway enrichment was assessed by GSEA desktop software using Hallmarks or KEGG gene sets.^[^ ^73^ ^]^ Customized R scripts were used to generate visualizations.

### Statistical Analysis

The statistical significance of differences between two groups was evaluated by two‐tailed Student's *t*‐test, while differences in multiple groups were analyzed by one‐way ANOVA or two‐way ANOVA in GraphPad Prism software. *p* values < 0.05 were considered statistically significant.

## Conflict of Interest

The authors declare no conflict of interest.

## Author Contributions

S.L., P.D., and Z.Y. contributed equally to this work. S.L., P.D., and J.T. conceived, designed, and supervised the study. Most of the in vitro and in vivo experiments were performed by S.L. and P.D. S.L. and P.D. analyzed and interpreted the data (e.g., statistical analysis, biostatistics, computational analysis). Z.Y. supervised the study and performed some in vitro experiments and helped in revised the manuscript. J.G., Y.H., R.X., J.Y., X.Z., Y.S., and P.W. provided material and technical support and helped in parts of involved experiments. R.G. and Y.H. conducted the assessment of organ toxicities in mouse models after combination treatment. J.H.H, J.Y.C., P.G., Q.Y., B.‐T.T., Q.J., X.X., Y.X., J.C., and Y.H. supervised the study and contributed to the manuscript writing. S.L., P.D., and J.T. wrote and revised the manuscript. All the authors reviewed the manuscript and gave their consent to publish this study.

## Supporting information



Supporting Information

## Data Availability

The raw and processed data of RNA‐Seq were deposited in the NCBI's Gene Expression Omnibus database under GSE247622.
