# Supplementary material for: CDC7 Inhibition Potentiates Antitumor Efficacy of PARP Inhibitor in Advanced Ovarian Cancer
Source: Adv Sci (Weinh). 2024 Oct 16;11(45):2403782. doi: 10.1002/advs.202403782 (PMC11615783; doi:10.1002/advs.202403782)

Supporting Information

**Title** CDC7 Inhibition Potentiates Antitumor Efficacy of PARP Inhibitor in Advanced Ovarian Cancer

Shini Liu^1,2#^, Peng Deng^1,3#^, Zhaoliang Yu^4#^, Jing Han Hong^5^, Jiuping Gao^1^, Yulin Huang^1^, Rong Xiao^1^, Jiaxin Yin^1^, Xian Zeng^1^, Yichen Sun^6^, Peili Wang^1^, Ruizi Geng^7^, Jason Yongsheng Chan^8^, Peiyong Guan^9^, Qiang Yu^5,9^, Bin-Tean Teh^5,8,9^, Qingping Jiang^10^, Xiaojun Xia^1^, Ying Xiong^1^, Jianfeng Chen^1^, Yongliang Huo^7^ and Jing Tan^1, 8, 11*^


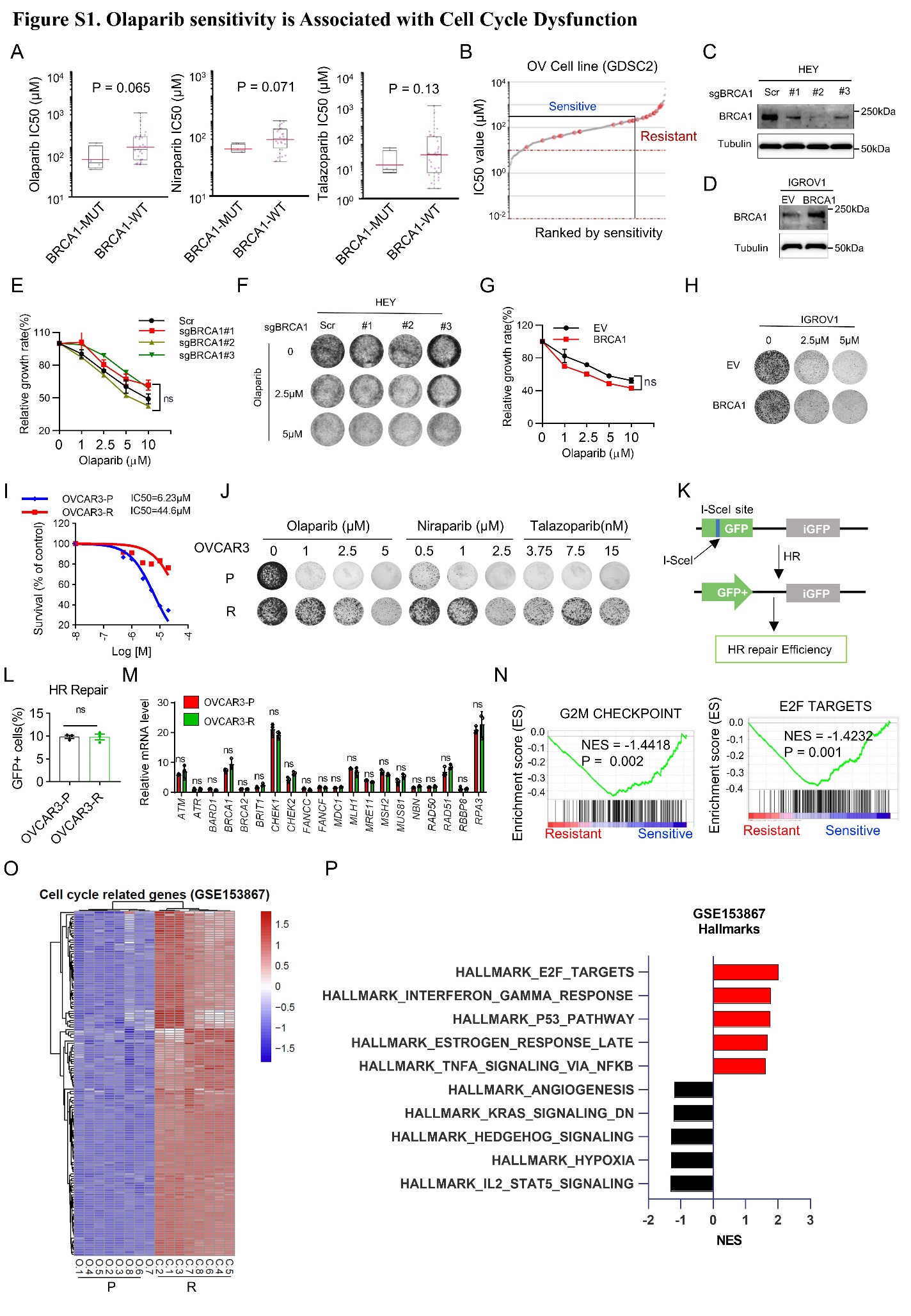


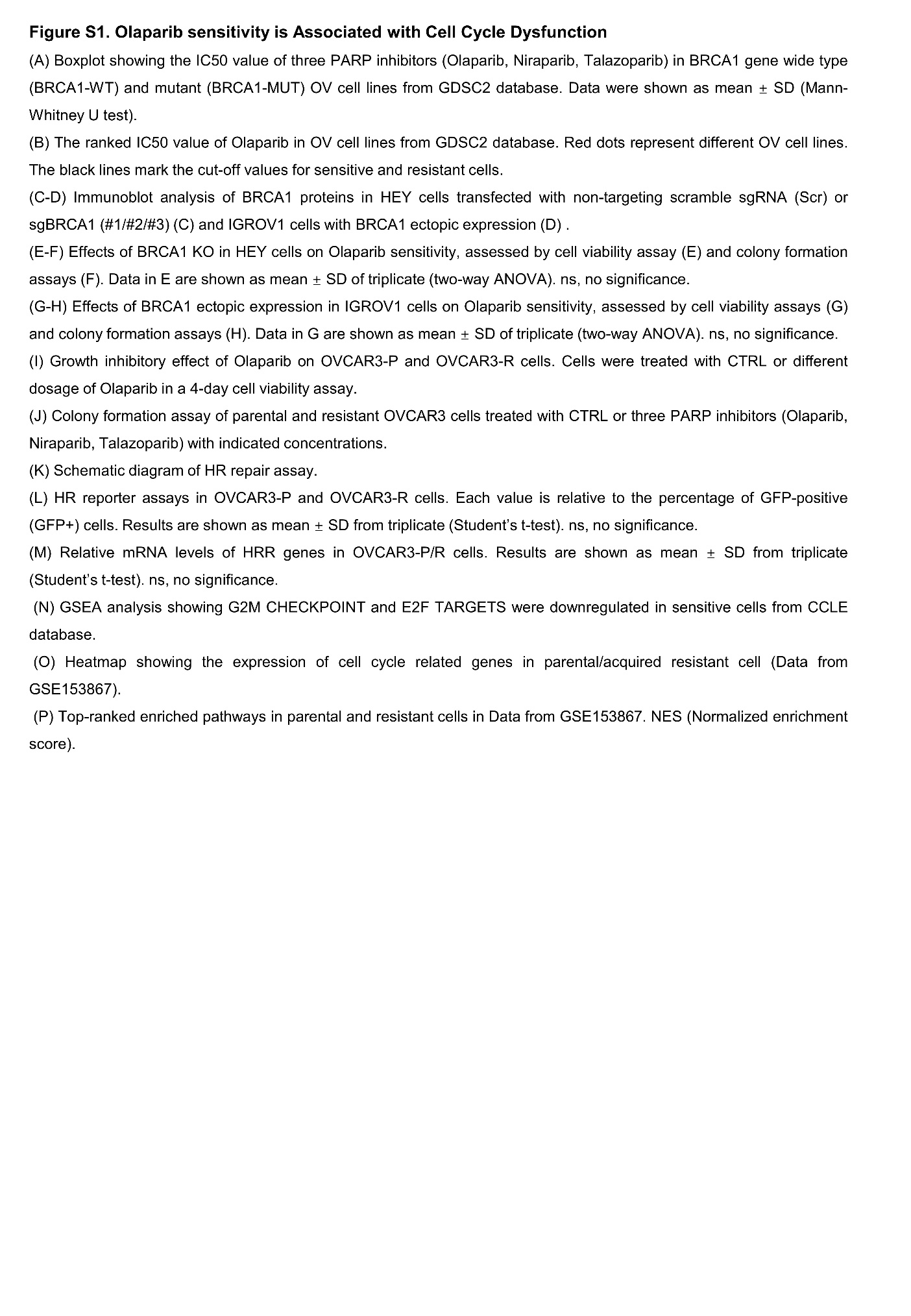


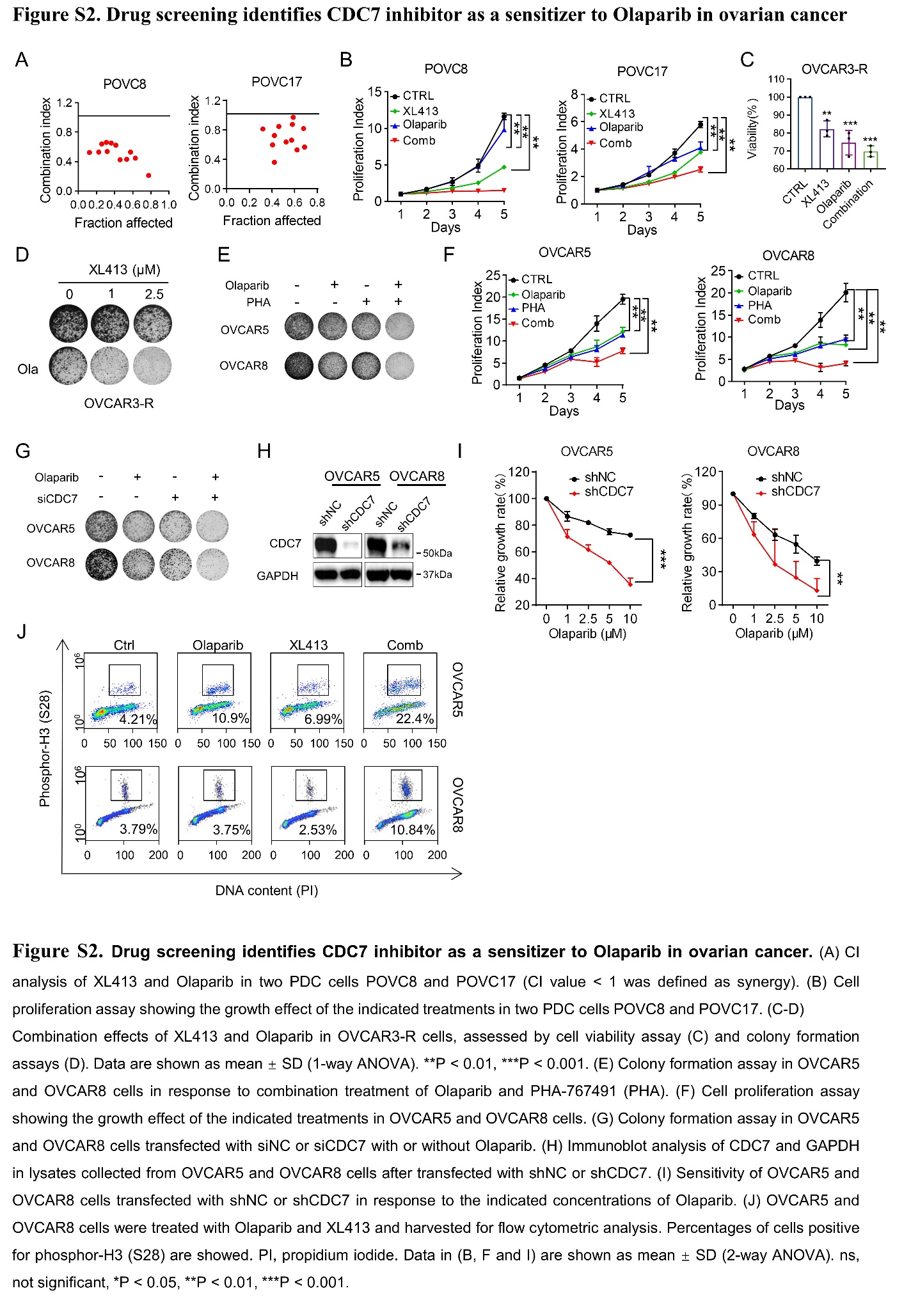


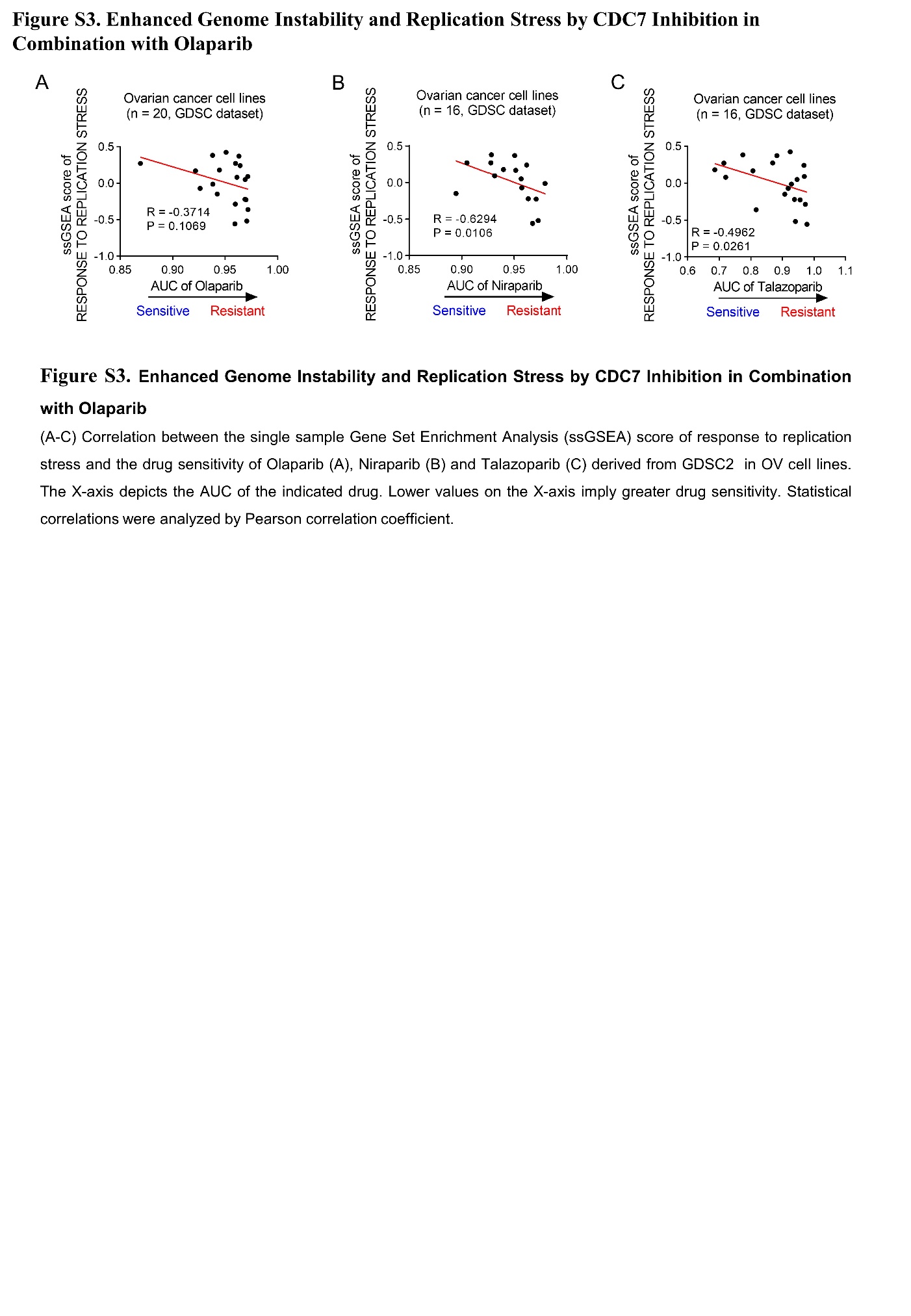


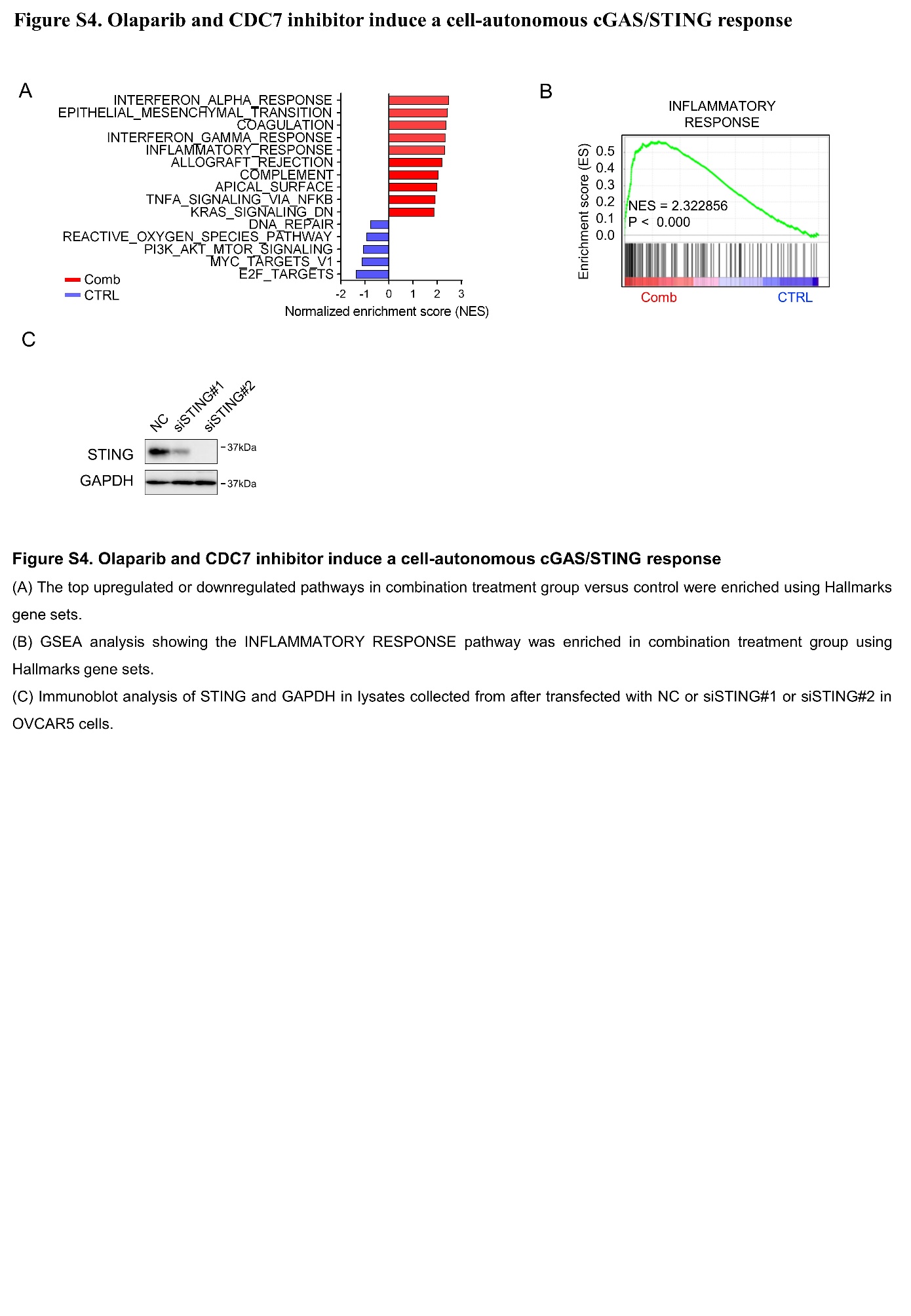


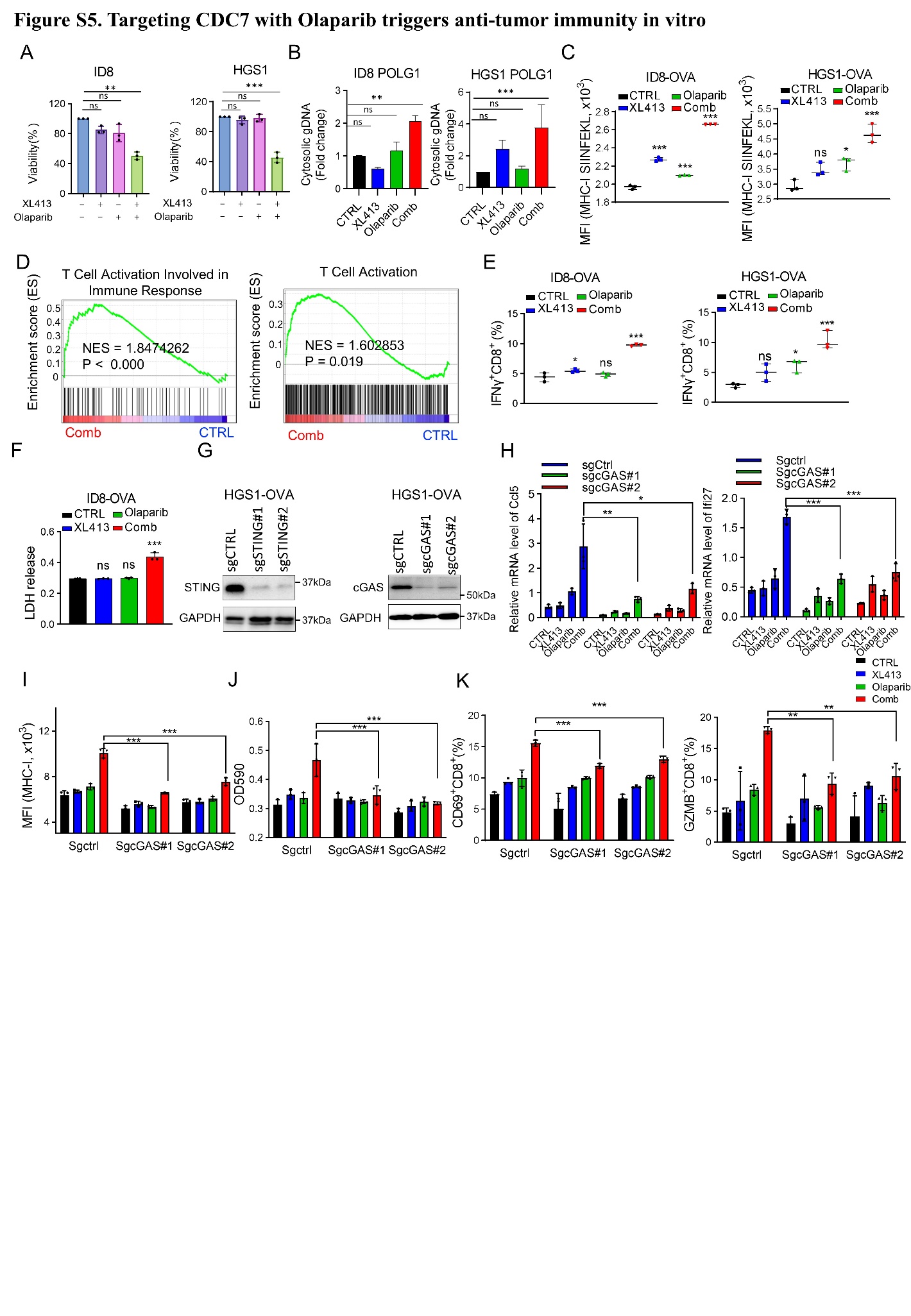


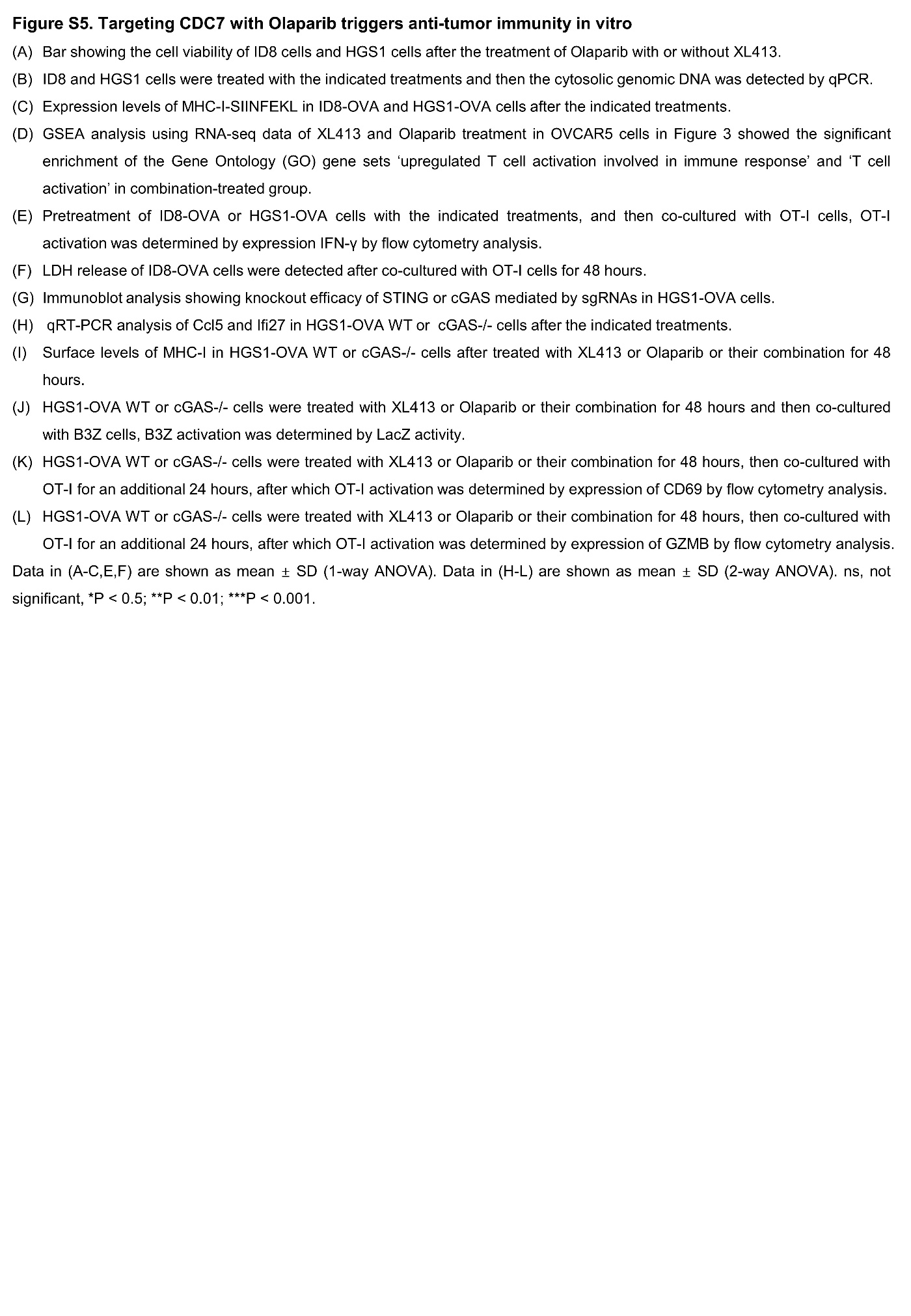


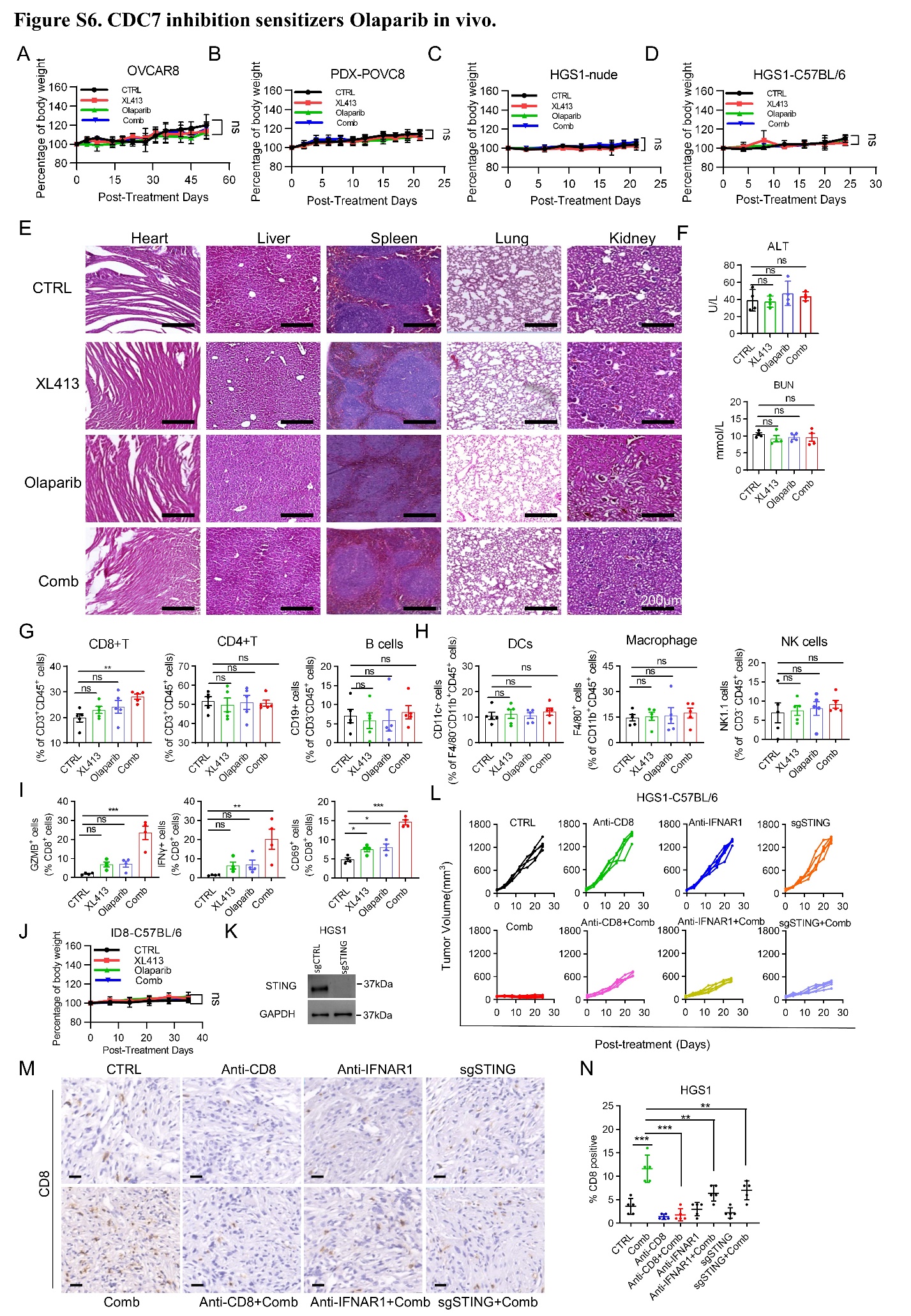


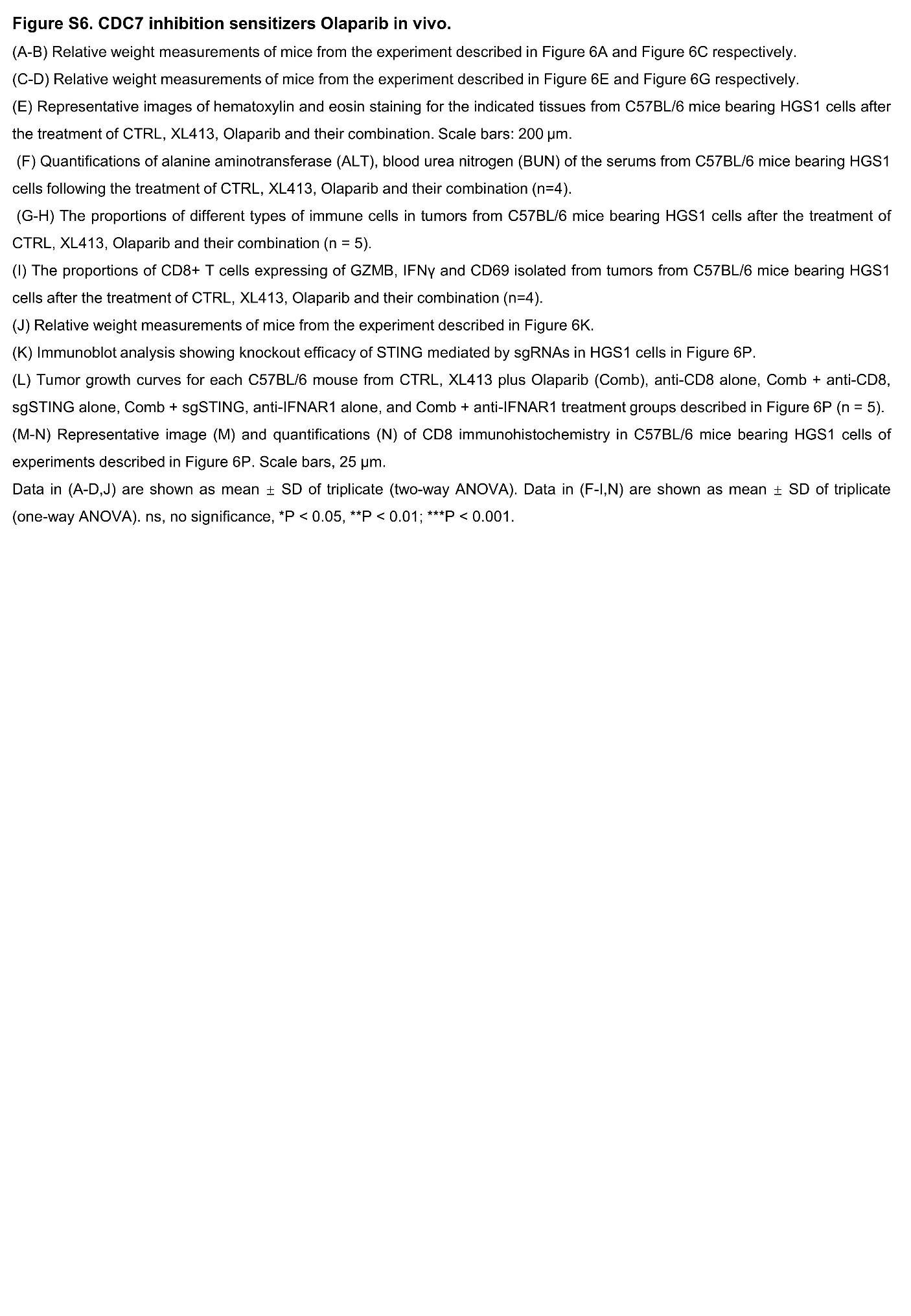


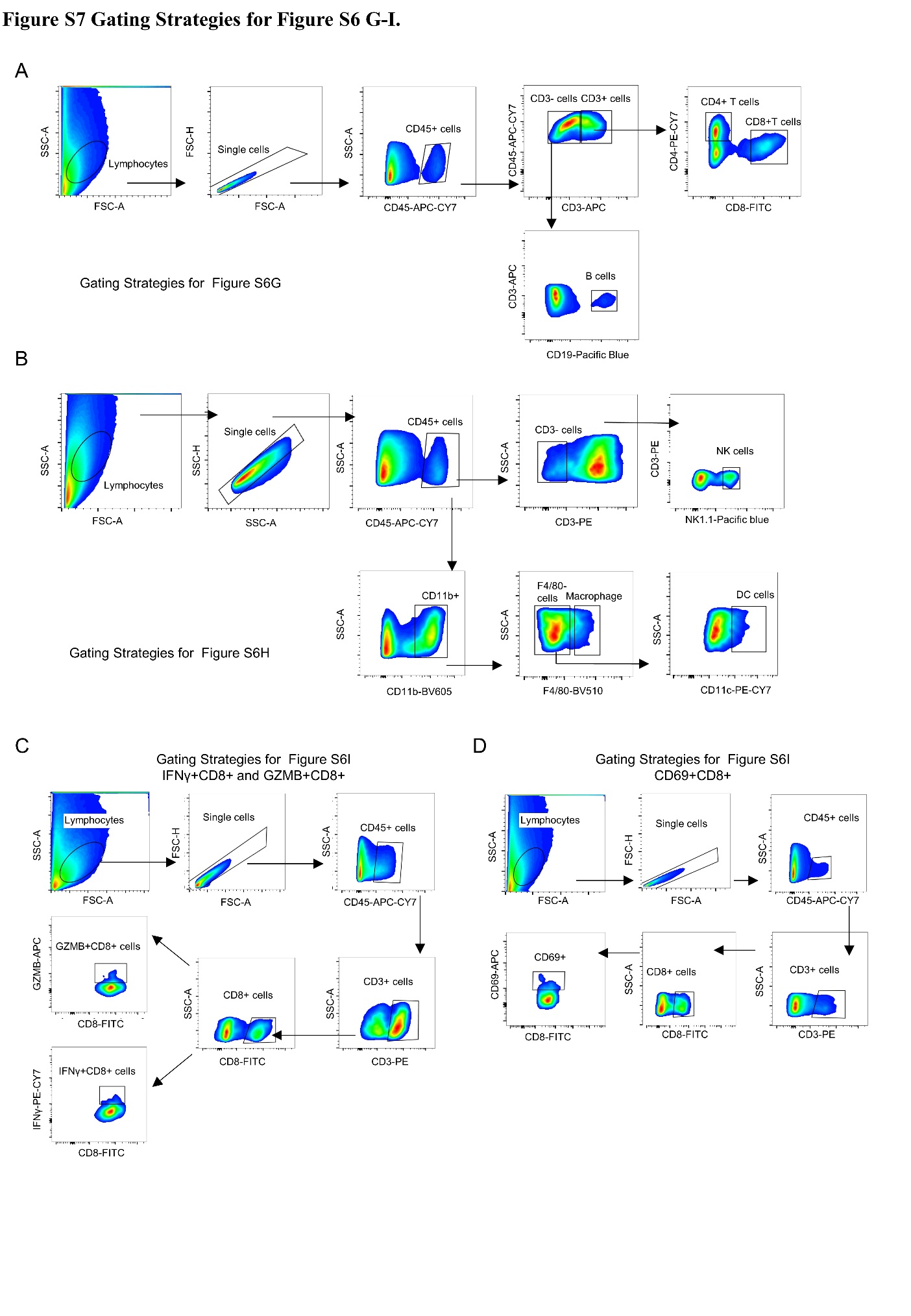


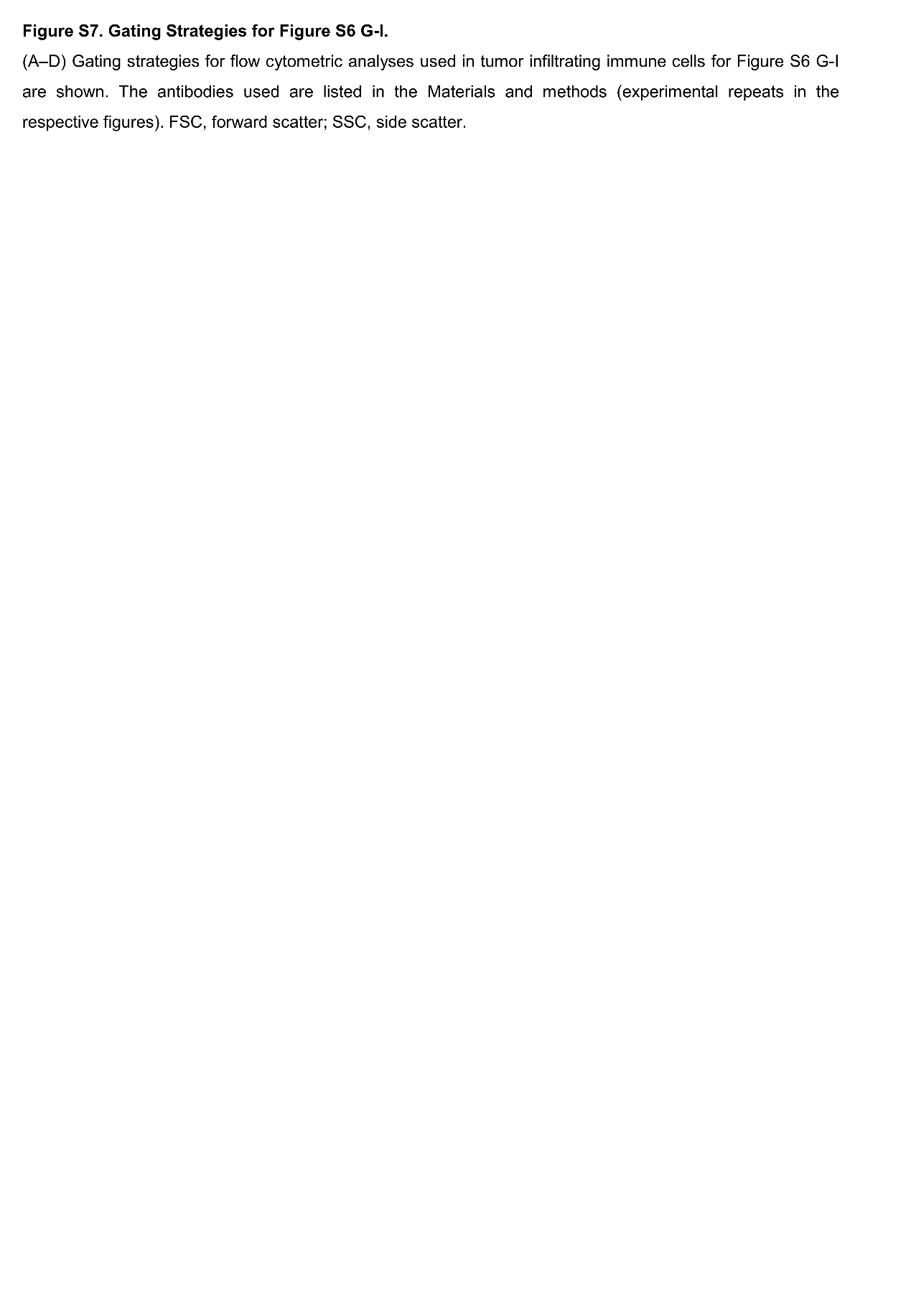


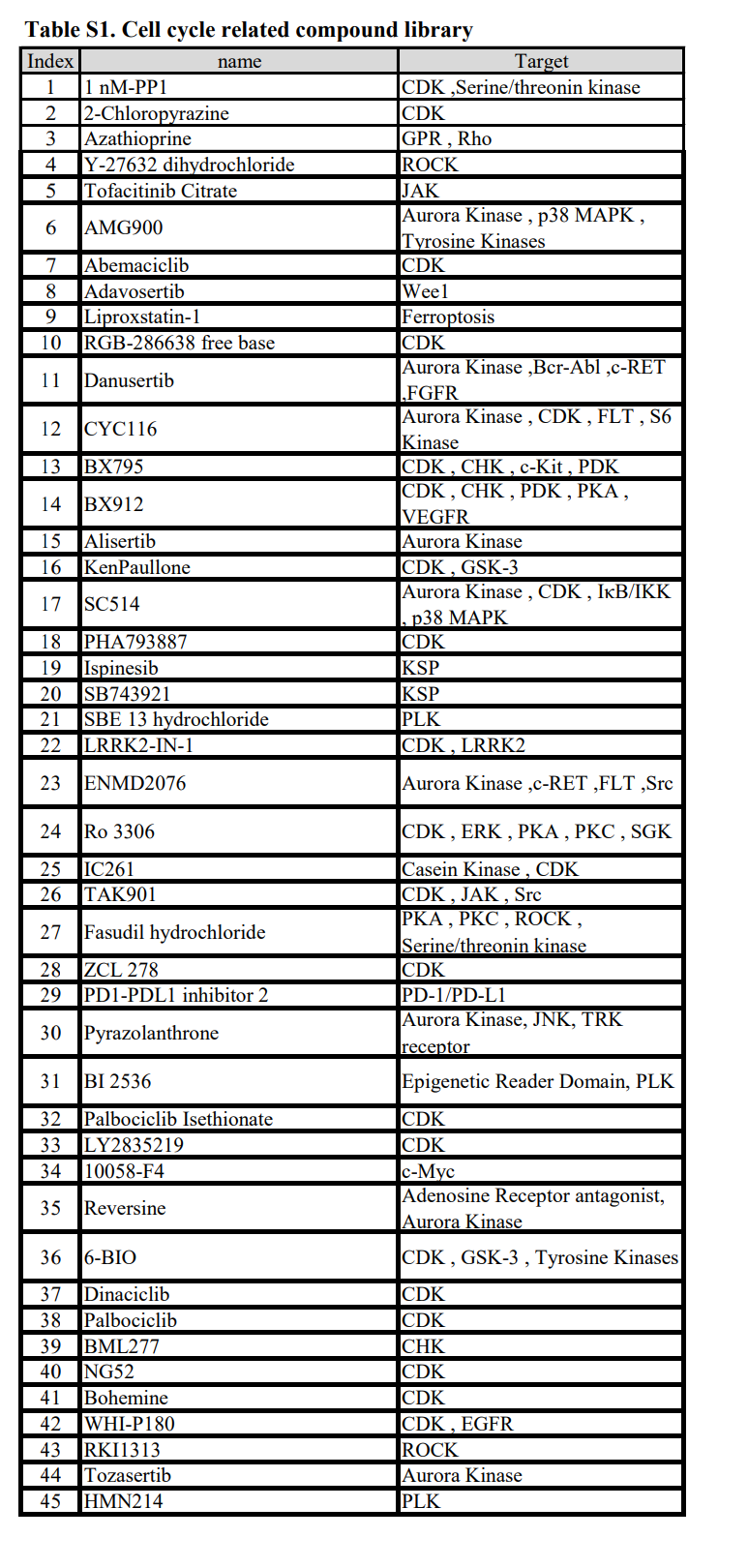


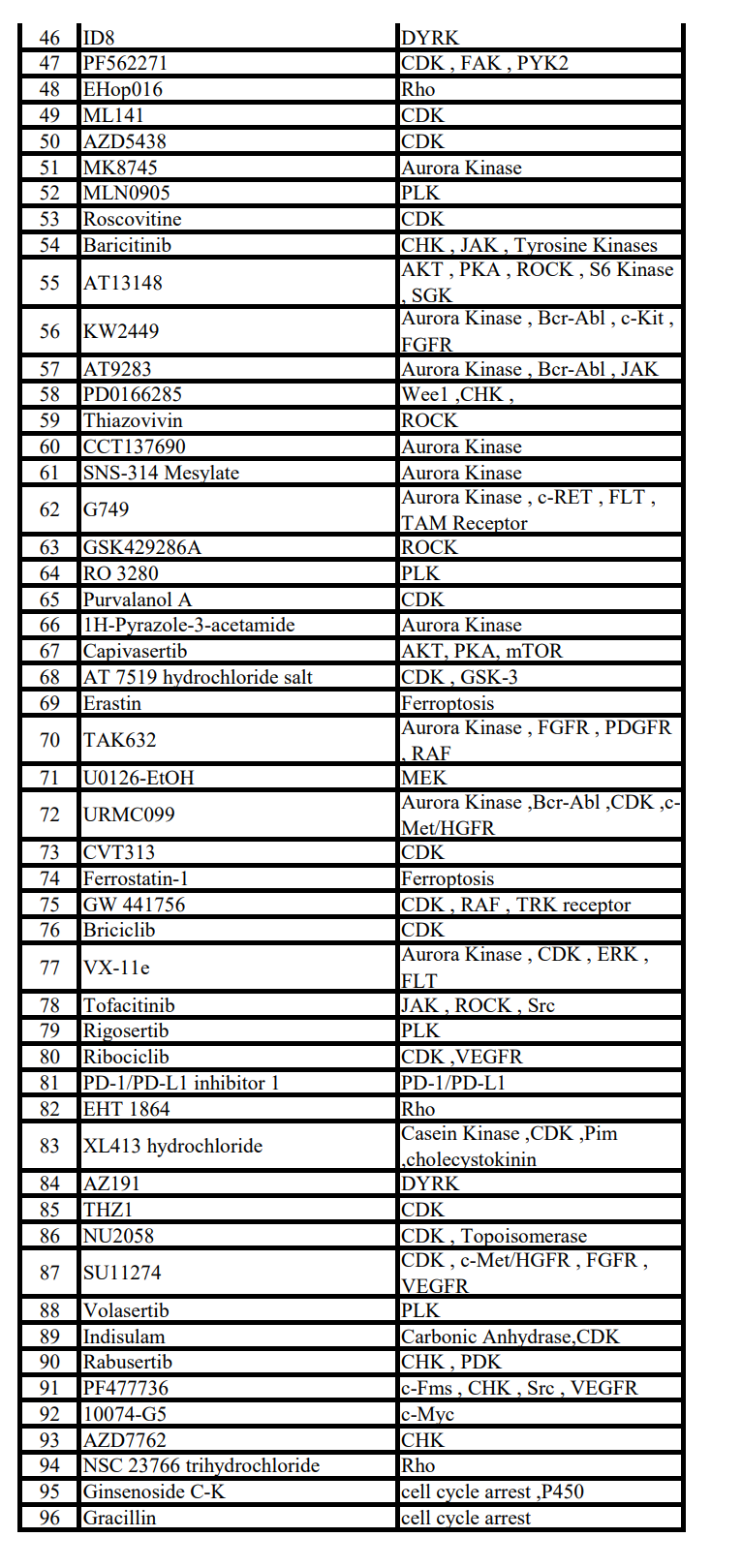


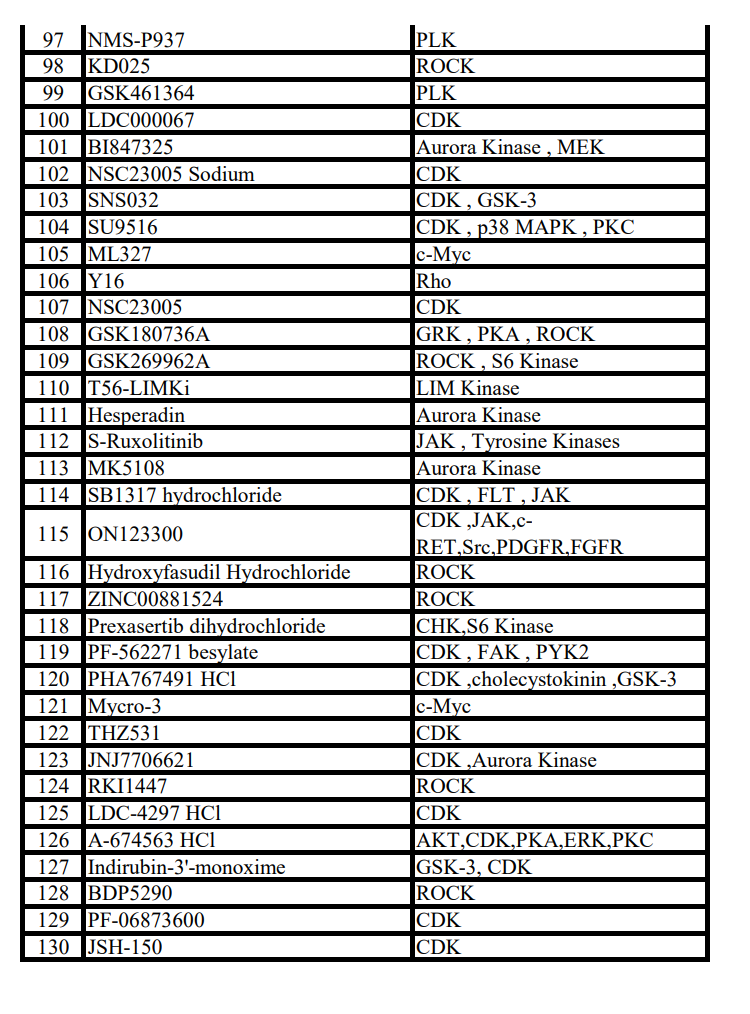


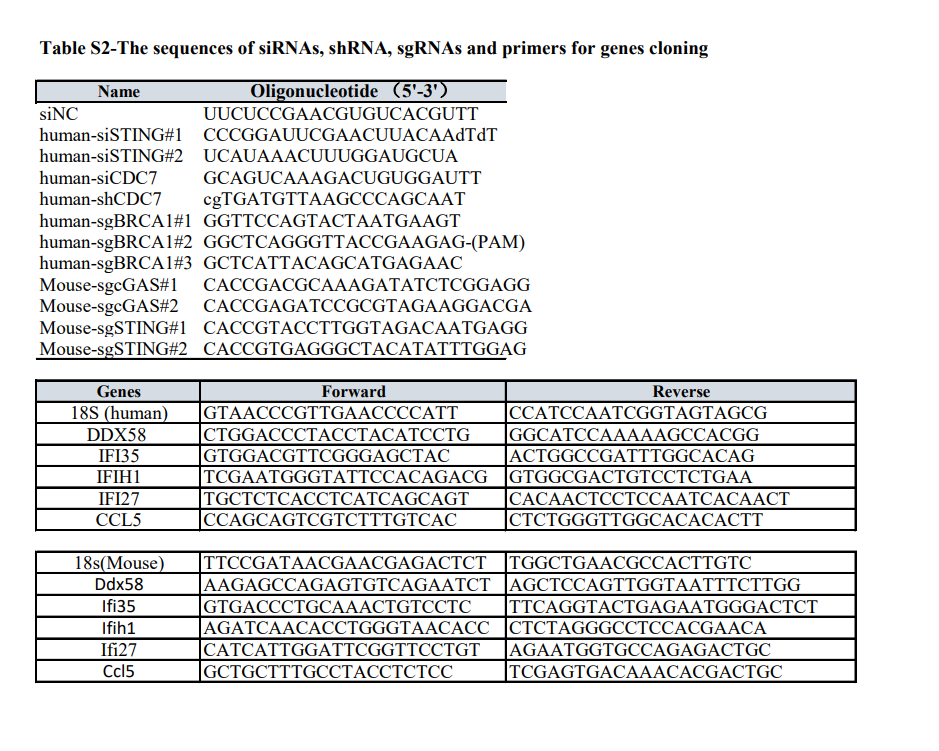

Supplement: Supplementary file 1 — Supporting Information [file ADVS-11-2403782-s001.docx]
